# Comparative Decomposition of Humans and Pigs: Soil Biogeochemistry, Microbial Activity and Metabolomic Profiles

**DOI:** 10.3389/fmicb.2020.608856

**Published:** 2021-01-13

**Authors:** Jennifer M. DeBruyn, Katharina M. Hoeland, Lois S. Taylor, Jessica D. Stevens, Michelle A. Moats, Sreejata Bandopadhyay, Stephen P. Dearth, Hector F. Castro, Kaitlin K. Hewitt, Shawn R. Campagna, Angela M. Dautartas, Giovanna M. Vidoli, Amy Z. Mundorff, Dawnie W. Steadman

**Affiliations:** ^1^Department of Biosystems Engineering and Soil Science, The University of Tennessee, Knoxville, Knoxville, TN, United States; ^2^Department of Chemistry, The University of Tennessee, Knoxville, Knoxville, TN, United States; ^3^Biological and Small Molecule Mass Spectrometry Core, Department of Chemistry, The University of Tennessee, Knoxville, Knoxville, TN, United States; ^4^Department of Anthropology, The University of Tennessee, Knoxville, Knoxville, TN, United States

**Keywords:** soil microbiology, carcass, forensic taphonomy, soil biogeochemistry, metabolomics, lipidomics, human decomposition, forensic anthropology

## Abstract

Vertebrate decomposition processes have important ecological implications and, in the case of human decomposition, forensic applications. Animals, especially domestic pigs (*Sus scrofa*), are frequently used as human analogs in forensic decomposition studies. However, recent research shows that humans and pigs do not necessarily decompose in the same manner, with differences in decomposition rates, patterns, and scavenging. The objective of our study was to extend these observations and determine if human and pig decomposition in terrestrial settings have different local impacts on soil biogeochemistry and microbial activity. In two seasonal trials (summer and winter), we simultaneously placed replicate human donors and pig carcasses on the soil surface and allowed them to decompose. In both human and pig decomposition-impacted soils, we observed elevated microbial respiration, protease activity, and ammonium, indicative of enhanced microbial ammonification and limited nitrification in soil during soft tissue decomposition. Soil respiration was comparable between summer and winter, indicating similar microbial activity; however, the magnitude of the pulse of decomposition products was greater in the summer. Using untargeted metabolomics and lipidomics approaches, we identified 38 metabolites and 54 lipids that were elevated in both human and pig decomposition-impacted soils. The most frequently detected metabolites were anthranilate, creatine, 5-hydroxyindoleacetic acid, taurine, xanthine, *N*-acetylglutamine, acetyllysine, and sedoheptulose 1/7-phosphate; the most frequently detected lipids were phosphatidylethanolamine and monogalactosyldiacylglycerol. Decomposition soils were also significantly enriched in metabolites belonging to amino acid metabolic pathways and the TCA cycle. Comparing humans and pigs, we noted several differences in soil biogeochemical responses. Soils under humans decreased in pH as decomposition progressed, while under pigs, soil pH increased. Additionally, under pigs we observed significantly higher ammonium and protease activities compared to humans. We identified several metabolites that were elevated in human decomposition soil compared to pig decomposition soil, including 2-oxo-4-methylthiobutanoate, sn-glycerol 3-phosphate, and tryptophan, suggesting different decomposition chemistries and timing between the two species. Together, our work shows that human and pig decomposition differ in terms of their impacts on soil biogeochemistry and microbial decomposer activities, adding to our understanding of decomposition ecology and informing the use of non-human models in forensic research.

## Introduction

Carrion decomposition is a critical component in biogeochemical cycling in all ecosystems. In terrestrial ecosystems, animals left to decompose on the soil surface are a nutrient and moisture-rich resource, creating a “hot spot” of enhanced biological activity (Keenan et al., 2018b). This ultimately serves to increase biodiversity and heterogeneity across landscapes. These decomposition hot spots have profound effects on the local soils, microorganisms, and plants (Towne, 2000; Carter et al., 2007; Bump et al., 2009; Parmenter and MacMahon, 2009; Yang et al., 2010; Barton et al., 2012, 2013a,b; Cobaugh et al., 2015; van Klink et al., 2020). Increases in ammonium, dissolved organic carbon, dissolved organic nitrogen, and phosphate are routinely documented (Cobaugh et al., 2015; Barton et al., 2016; Fancher et al., 2017; Keenan et al., 2018a). Concomitant with this is temporal succession of local biological communities, including arthropods, plants, microbes and microfauna (Schoenly and Reid, 1987; Barton et al., 2013a; Macdonald et al., 2014; Cobaugh et al., 2015; Finley et al., 2016; Szelecz et al., 2016; Keenan et al., 2018a; Taylor et al., 2020).

While certain patterns have emerged from these studies of carcass decomposition, it is also generally noted that there can be wide variability in soil physicochemical responses. For example, soil pH in decomposition-impacted soils does not exhibit predictable patterns: Some studies reported decreased pH associated with surface decomposition (Towne, 2000; Aitkenhead-Peterson et al., 2012), others report an increase (Benninger et al., 2008; Meyer et al., 2013; Keenan et al., 2018b; Szelecz et al., 2018), and still others found no significant change (Cobaugh et al., 2015; Fancher et al., 2017). This variability in soil pH is important because soil pH controls not only the soil chemistry, it is also a key driver of bacterial community structure generally (Lauber et al., 2009), ultimately influencing the composition of the decomposer community.

Understanding patterns and processes of mammalian decomposition has important forensic applications. Forensic taphonomy research often focuses on using patterns of decomposition to improve estimates of postmortem interval (PMI) or time since death estimations (e.g., Carter and Tibbett, 2003; Tibbett and Carter, 2003; Metcalf et al., 2013; Pechal et al., 2013; Hauther et al., 2015; Johnson et al., 2016; von der Lühe et al., 2017). Because of challenges in obtaining, and/or restrictions on use of, human cadavers, many forensic taphonomy studies use animal carcasses as proxies. Therefore, much of our knowledge of decomposition timing and processes have relied on various animal carcasses, including pigs (Hopkins et al., 2000; Wilson et al., 2007; Howard et al., 2010; Meyer et al., 2013; Pechal et al., 2013; Forger et al., 2019; Matuszewski et al., 2020), mice (Metcalf et al., 2013; Lauber et al., 2014), rats (Carter et al., 2010), dogs (Reed, 1958), and other vertebrate wildlife (Towne, 2000; Parmenter and MacMahon, 2009; Macdonald et al., 2014; Risch et al., 2020). Domestic pigs (*Sus scrofa*) are often cited as the most useful analog for humans in decomposition studies, given their physiological and anatomical similarities, including mass, hairiness, and pigmentation (Schoenly et al., 2007; Matuszewski et al., 2020). Studies using pigs often have greater replication, and have been formative in establishing proof-of-concept for forensic methods (Matuszewski et al., 2020). In general, studies have revealed that pigs and humans host similar postmortem insect communities (Schoenly et al., 2007; Wang et al., 2017; Matuszewski et al., 2020). Species similarities in postmortem soil and decomposer dynamics are less well understood. Pigs and humans are both monogastric omnivores, having a relatively similar gut microbiome composition (Ley et al., 2008) in comparison to carnivores or herbivores. However, different species of mammalian carcasses each have their own unique composition: For example, pigs have a greater moisture content while humans have greater nitrogen content (Carter et al., 2007). Additionally, pigs have higher levels of total saturated fatty acids compared to humans, contributing to differences in adipocere formation between pigs and humans (Notter et al., 2009). There is also evidence that the suite of volatile organic compounds responsible for decomposition odors differs between species (Vass et al., 2008); a fact that is exploited by canines trained in human remains detection.

Despite the evidence that decomposition processes may differ between different species, most of the existing studies have confounding variables that hamper interpretation (Matuszewski et al., 2020). Only a few studies have made direct comparisons: one study compared 10 non-human vertebrates ranging in mass from 6 g (deer mouse) to 13 kg (mule deer), revealing that mass loss rates were variable between species and not correlated to initial body mass (Parmenter and MacMahon, 2009). Another study comparing buried skeletal muscle tissue from four mammals (including humans) showed that while soil nutrient enrichment patterns associated with decomposition followed the same overall temporal patterns, there were differences in nitrogen flux; namely soil ammonium concentrations associated with porcine and bovine tissues were twice as high as human tissue (Stokes et al., 2013). To address the question of whether pigs can be used as proxies in forensic taphonomy research, a study was undertaken at the University of Tennessee Anthropology Research Facility (ARF). Over three seasonal trials which directly compared humans, pigs, and rabbits, the study quantified gross morphological changes via the Total Body Score method (Megyesi et al., 2005). This study revealed that pigs do not exhibit the same decomposition patterns as humans, and noted that humans had greater variability, scavenging, and mummification compared to animals (Dautartas et al., 2018; Steadman et al., 2018). This finding was corroborated by a similar study conducted in an arid environment, which also concluded that humans and pigs displayed differential decomposition (Connor et al., 2018).

Given that humans and pigs have differential morphological decomposition patterns, the objective of our study was to determine if human and pig decomposition have similar effects on soil. Our null hypothesis was that because of their similar mass, the decomposition of these two species under identical environmental conditions should have similar effects on soil physicochemistry, microbial activity, biogeochemistry and decomposition products. To address our hypothesis, we measured several soil physicochemical parameters and indicators of microbial activity, including respiration, protease activity and untargeted metabolomic profiles during two comparative human and pig decomposition trials conducted at the ARF (reported in Dautartas et al., 2018; Steadman et al., 2018). Untargeted metabolomics has emerged as a powerful tool in systems biology, focusing on the identification and quantitation of low molecular weight metabolites present in a system (<1,000 Da). More recently, it has been applied to characterize microbial communities from various environmental samples, including soil (e.g., Randewig et al., 2019; Withers et al., 2020). Here, we applied an untargeted metabolomics and lipidomics approach to soil collected in close proximity to decomposing carcasses to characterize decomposition products. Together, our work provides a direct comparison of human and pig decomposition in terms of their impacts on soil biogeochemistry and microbial decomposer communities, adding to our understanding of decomposition ecology and providing important information for evaluating the use of non-human analogs in forensic research.

## Materials and Methods

### Study Design

Two comparative decomposition trials conducted at the University of Tennessee Anthropology Research Facility (ARF) were the focus of this study, conducted in the summer of 2014 and the winter of 2014–2015. The ARF, located in Knoxville, Tennessee (35° 56′ 28′′ N, 83° 56′ 25′′ W), is a nearly 3-acre outdoor laboratory dedicated to the study of human decomposition. The site is a temperate deciduous forest with well-drained fine textured soils (Damann et al., 2012). The textural class of the soils at this site based on particle size analysis was silt loam (>55% silt) (L. S. Taylor, *personal comm*.).

The human subjects were donations to the University of Tennessee Forensic Anthropology Center^1^. As no living human subjects were involved, this work was exempt from review by the University of Tennessee Institutional Review Board. No preference was employed for donor sex, age, or ancestry. The University of Tennessee protocol for accepting donations ensured the individuals did not have communicable diseases. The bodies were not autopsied or embalmed, nor had signs of external trauma. All subjects were placed in a 4°C morgue cooler for at least 24 h before placement to equalize body temperature. The human donors consisted of six adult females and four males, with weights ranging from 53 to 107 kg, and all died of natural causes. Ten pig (*S. scrofa*) carcasses were obtained from a local farm near Knoxville, Tennessee. The summer trial used three female and two male pigs, ranging from 40 to 59 kg in weight. The winter trial used two female and three male pigs, ranging from 47 to 57 kg. A veterinarian euthanized the animals via injection; all protocols for animal handling and euthanasia were approved by the University of Tennessee Institutional Animal Care and Use Committee (IACUC). Full details on the donors, carcasses, and euthanasia protocols are provided in Dautartas et al. (2018).

The experimental design has been described in detail previously (Dautartas et al., 2018; Steadman et al., 2018). In each trial, five human subjects and five pig (*S. scrofa*) carcasses were placed on virgin soils that had not previously been used for decomposition experiments at the ARF. They were placed in a randomized block design, along with domestic rabbit (*Oryctolagus cuniculus*) carcasses which were not included as part of our current study (Supplementary Figure 1). Carcasses were placed with a minimum of 3 m between each carcass. Internal temperatures for human donors were monitored using Tiny Tag data loggers (Gemini Data Loggers, United Kingdom) with probes inserted rectally. Soil temperatures for both pigs and humans were recorded with Tiny Tag data loggers inserted 5–10 cm into the soil immediately adjacent to the donors. Ambient temperature and humidity were recorded hourly using Tiny Tag loggers suspended from nearby trees, and used to calculate Accumulated Degree Days (ADD) as described in Dautartas et al. (2018). Morphological changes and tissue loss of the carcasses was scored using the Total Body Score (TBS) (Megyesi et al., 2005).

### Soil Sampling

Soil samples were collected prior to carcass placement, and then from the area immediately adjacent to each carcass during decomposition. Due to physical site constraints that limited access to soils (terrain and thick vegetation), the summer trial included four human subjects and three pigs; the winter trial included five humans and five pigs. Soils were additionally collected from control sites that were at least 5 m from the carcasses and had not been previously exposed to decomposition. Five meters was reasonably assumed to be beyond the zone of influence of the carcasses: Past research at the ARF and nearby sites has revealed minimal (<1 m) lateral translocation of decomposition products or effects on soil biota beyond the area of visible decomposition fluid saturation (Keenan et al., 2018a, 2019); other researchers have also reported that soil communities were affected up to 1 m away from human cadavers, but unaffected at 5 m (Singh et al., 2018). For the summer trial, samples were collected weekly for 5 weeks, which corresponded to approximately 400 ADD and TBS of approximately 28 for pigs and 24 for humans (Dautartas et al., 2018). For the winter trial, weekly samples were collected for the first 2 months, when decomposition was most variable. Following that, soils were collected monthly for a total of 21 weeks, which corresponded to approximately 600 ADD and TBS of 15 for pigs and 25 for humans (Dautartas et al., 2018). Soils were collected from the top 0–5 cm using sterile 15 mm plastic corers, following the method of Cobaugh et al. (2015). At each sampling time point, approximately 20 core samples were taken from the area around each donor which was visibly discolored by decomposition fluids, up to 30 cm away from each donor. Core sample locations were selected throughout the visible decomposition zone to ensure uniform coverage of the area (i.e., equal number of cores were taken from the head, torso, and limb areas). The core samples were composited in a sterile Whirl-pak^®^ bag, and transported back to the lab for immediate processing. Soils were thoroughly homogenized prior to laboratory analyses, and subsamples stored at −20°C for enzyme assays and −80°C for metabolomics analyses.

### Soil Physicochemical Analyses

Gravimetric soil moisture was determined by oven-drying soils at 105°C for at least 48 h. Soil pH was measured at 20°C using a 1:2 soil to deionized water (dH_2_O) slurry and an Orion multiparameter meter (Orion Star A329, Thermo Scientific). Water soluble nutrients were extracted from soils by mixing soils 1:5 in dH_2_O and shaking for 4 h at 170 rpm. Samples were centrifuged at 188 rcf at 20°C for 25 min to settle soils, and supernatants were filtered through grade GF/F glass microfiber filters (Whatman^TM^) to remove suspended particulates. Extracts were stored at −20°C until analysis. Ammonium concentrations in soil extracts were quantified following a microplate protocol after a 2 h incubation, with minor modifications (Rhine et al., 1998). The ammonium standard [(NH_4_)_2_SO_4_] was dissolved in dH_2_O to account for potential matrix effects. In addition, 70 μl (instead of the 50 μl specified in original protocol) of each soil extract or standard were pipetted into the microplate, and 50 μl of deionized water was used (instead of 100 μl). Nitrate concentrations were determined (in triplicate) using a colorimetric method (Doane and Horwáth, 2003) using 50 μl of the NO_3_^–^ color reagent and 70 μl of the soil extracts in a 96-well plate. Absorbance values were measured using a plate reader at 543 nm after incubating for at least 10 min at room temperature (Doane and Horwáth, 2003). Data analysis of physicochemical parameters was done in R v3.2.0 using R Studio v0.99.491. An initial screen with a mixed model showed that time was a significant factor, so subsequent analyses focused on comparing the treatments for each date separately. ANOVAs followed by a *post hoc* TukeyHSD comparison test were used to identify significant differences between treatments for each date.

### Soil Biological Activity

Soil respiration rates over 24 h were determined by incubating 10 g (wet weight) soils in sealed 70 ml vials fitted with septa. At the beginning and end of incubation, 0.25 ml headspace samples were taken in duplicate and manually injected and read on a LiCor LI-820 CO_2_ Analyzer (LiCor Inc., Lincoln, NE, United States). As a proxy for protein degradation activity, leucine amino peptidase activity was measured, according to Bell et al. (2013). Briefly, 2.75 g of soil was slurried with 91 ml Tris buffer (pH 6.7) in a blender. L-leucine-7-amido-4-methylcoumarin hydrochloride (200 μl) was added to the slurries in a 96-deep-well plate. Plates were incubated for 3 h at 20°C, then centrifuged to settle soil particles. 250 μl of the supernatant was transferred to a new 96-well plate and read on a Synergy H1 plate reader (BioTek, Winooski, VT, United States). Data analysis of microbial activity rates was performed as described for soil physicochemical parameters.

### Metabolomics and Lipidomics

#### Extraction Method

Metabolomics and lipidomics were only performed on soil samples from the winter trial, as these soils had been appropriately flash-frozen and preserved at −80°C. Unfortunately, soil samples from the summer trial had been stored at −20°C, which is not recommended for long term storage of samples for metabolomics (Pinto et al., 2014; Hernandes et al., 2017), and therefore we were not able to perform metabolomic and lipidomic analysis for the summer trial. For the winter trial samples, the entirety of the extraction process was performed at 4°C unless otherwise stated. Soil samples were crushed with mortar and pestle under liquid nitrogen and weighed (approximately 100 mg) into individual 2 ml microcentrifuge tubes. To each tube, 1.3 ml of extraction solvent consisting of 40:40:20 HPLC grade methanol, acetonitrile, and water with 0.1 M formic acid (Thermo Fisher Scientific, Waltham, MA, United States) was added (Rabinowitz and Kimball, 2007). Soil particles were suspended by vortexing before extraction was carried out for 20 min while being shaken in an orbital platform shaker (Bellco, Vineland, NJ, United States). Following extraction, samples were centrifuged for 5 min at 16,100 rcf and the supernatant was removed and combined with the supernatant from the first extraction in new microcentrifuge tubes. The remaining soil was resuspended in 200 μl of extraction solvent and incubated for another 20 min while being shaken. Following extraction, samples were centrifuged for 5 min at 16,100 rcf before the supernatant was again transferred to the same microcentrifuge tubes. The samples were evaporated to dryness under a stream of nitrogen gas. The resulting dried residue was resuspended in 300 μl of sterile Milli-Q^®^ grade water and transferred to autosampler vials for subsequent mass spectrometric analysis. Lipidomics extractions followed a modified version of the procedure described by Bligh and Dyer (1959). The method used two extraction solvents, 1:1 (v/v) 0.1 N hydrochloric acid: methanol (solvent 1), and 100% chloroform (solvent 2), which were added to approximately 50–100 mg of crushed soil samples. 800 μl of solvent 1 and 400 μl of solvent 2 were mixed and added to the soil samples. Samples were vortexed for 10 s and centrifuged at 16,100 rcf for 5 min. The chloroform layer was isolated and dried under nitrogen and resuspended in 9:1 (v/v) methanol: chloroform in autosampler vials prior to analysis.

#### Mass Spectrometry Methods

Mass spectrometry methods were adapted from Lu et al. (2010) for soil samples as we have described previously (Mueller et al., 2020). Autosampler vials were placed in autosampler trays (Ultimate 3000 RS Autosampler, Dionex, Sunnyvale, CA, United States) maintained at 4°C. A 10 μl aliquot from each vial was injected through a Synergi 2.5 μ reverse-phase Hydro-RP 100, 100 mm × 2.00 mm liquid chromatography column (Phenomenex, Torrance, CA, United States) maintained at 25°C. Chromatographic elution was ionized via electrospray ionization (Spray voltage: 2 kV, nitrogen sheath gas: 10, capillary temperature: 320°C) and introduced to an Exactive Plus Orbitrap mass spectrometer (Thermo Fisher Scientific, Waltham, MA, United States). LC-MS analysis was performed in negative ionization mode with a full-scan covering a window of 85 to 800 *m/z* from 0 to 9 min and 110 to 1,000 from 9 to 25 min. The resolution was set to 140,000 and the acquisition gain control target to 3e6. Solvent A was composed of 97:3 water: methanol with 10 mM tributylamine and 15 mM acetic acid. Solvent B consisted of methanol. The gradient was as follows: 0 to 5 min: 0% B, 5 to 13 min: 20% B, 13 to 15.5 min: 55% B, 15.5 to 19 min: 95% B, 19 to 25 min: 0% B using a constant flow rate of 200 μl min^–1^.

#### Metabolomics Data Processing and Analysis

Mass spectrometry data files generated by Xcalibur were converted to mzML format (Martens et al., 2011) using the Proteowizard package (Chambers et al., 2012). Sample non-linear retention time correction, metabolite identification, and chromatogram integration were performed using MAVEN (Melamud et al., 2010; Clasquin et al., 2012). Metabolites were manually selected based on known standards (±5 ppm mass tolerance and ≤1.5 retention time tolerance). Unidentified spectral features were annotated using MAVEN’s automatic peak detection algorithms with the settings as follows: Mass domain resolution was 10 ppm, time domain resolution was 10 scans, and EIC smoothing was 5 scans with 0.5 min peak grouping. Baseline smoothing was 5 scans and the top 80% of intensities were dropped from chromatogram for baseline calculation. Peak scoring was performed based on a trained classifier model looking for 4 minimum peaks per group, 5 minimum signal-to-noise, 5 minimum signal-to-blank, 10,000 minimum signal intensity, and 5 scan minimum peak width. Spectral features were normalized by soil dry weight. For each metabolite identified, relative intensity was calculated as the intensity in a given sample normalized to intensities in all samples. Relative intensities were uploaded to MetaboAnalyst online statistical analyzer for further analyses. Partial least squares-discriminant analysis (PLS-DA) and variable importance in projection (VIP) scores were calculated using MetaboAnalyst (Chong et al., 2019). MetaboAnalyst was also used to determine metabolic pathways using a human metabolic reference map that is available through KEGG (Kyoto Encyclopedia of Genes and Genomes). Heatmaps were generated in R v1.1.423 using ggplot2 (Wickham, 2009). The metabolomics profiling data is available in the MetaboLights database^2^ under study MTBLS2254.

## Results

### Ambient, Soil, and Internal Donor Temperatures

For the summer trial (June 2014), the mean ambient air temperature was 24.4°C. The ambient mean maximum was 38.5°C (day 4) and mean minimum was 15.0°C (day 34). Soil temperatures were recorded hourly from sensors placed in soil adjacent to human donors; the soil sensor associated with donor number 7 failed during the experiment, so these data were not used for analyses. The mean soil temperature for the study was 25.3°C (Supplementary Figure 2A). Soil temperatures in decomposition soils were slightly elevated in comparison with ambient temperatures during days 4 through 17. Soil temperatures reached a mean maximum of 33.9°C on study day 8, and a mean minimum of 19.1°C on study day 1. Temperature probes were placed inside human donors to measure internal temperature fluctuations during decomposition progression. Internal donor temperatures mirrored those of soil temperatures, diverging from ambient air temperatures. The internal temperature mean for the duration of the study was 26.1°C, and reached a mean maximum temperature of 39.8°C on study day 7 (1 day prior to mean maximum soil temperatures) (Supplementary Figure 2A). The internal mean minimum was 7.1°C at the beginning of the study (day 1).

For the winter trial (starting in December 2014), the mean ambient temperature was 4.7°C, with a mean maximum of 33.4°C, and mean minimum of −16.5°C. Soil temperatures closely followed ambient temperatures, although with less daily variation; the mean soil temperature over the course of the study was 6.2°C (Supplementary Figure 2B). Soil temperatures reached a mean maximum of 15.9°C on study day 123 (week 17) and minimum of −0.13°C on day 81 (week 12). Internal donor temperatures did not vary appreciably from either soil or ambient air temperatures; the overall study mean was 6.3°C. Internal donor temperatures reached a mean maximum of 27.7°C on study day 114 (week 16) and mean minimum of −2.8°C on day 82 (week 12) (Supplementary Figure 2B).

### Soil Physicochemistry

For the winter trial, all ten study plots were intended to be replicates and were in the same local area and on the same soil type. However, we retrospectively noted some variability in the response of several measured parameters, and that this variability was dependent on the location of the plot. Namely, six of the plots (three human and three pig) were on undisturbed forest soils with a visible O horizon and neutral pH (pH 6 to 7) (hereafter referred to as “lower site”). The other four plots (two human and two pig) were placed closer to a fence line (hereafter, “upper site”) where soils had been previously disturbed from construction activities nearby. These upper site soils were visibly lighter in color: They did not have an O horizon, were lower in organic matter, and had a higher pH at the start of the study (pH 7 to 8). In order to account for the confounding factor of location, we first tested all variables for a plot location effect. Where a significant effect was observed (for pH, LAP activity, and metabolite profiles), we split the dataset by location and analyzed the two sites separately. Where no location effect was observed (nitrate, ammonium, and respiration), the data were analyzed together.

**FIGURE 1:**
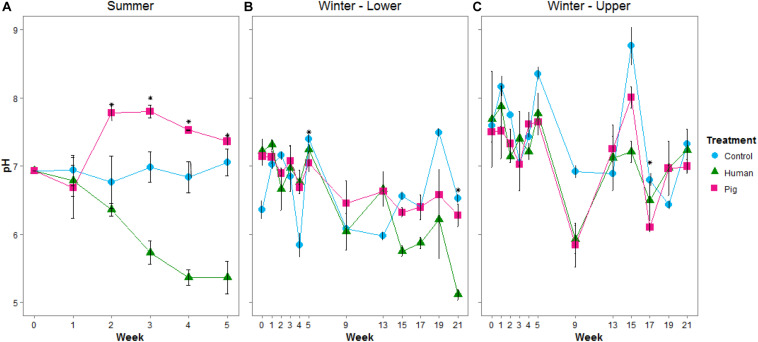
Mean pH of soils below decomposing humans and pigs during the summer **(A)** and winter **(B,C)** trials. The lower site **(B)** refers to undisturbed forest soil, while the upper site **(C)** had disturbed soil. Error bars show standard deviations. Asterisks indicate sample times with significant differences between the three treatments (*p* < 0.05).

During the summer trial, soils became more alkaline under pigs, and more acidic under humans starting at week 3 (approximately 438 ADD; Supplementary Table 1). At this time, pigs were slightly more decomposed, with a TBS of 29 compared to human TBS of 25 (Supplementary Table 1). pH differences continued for the duration of sampling (Figure 1A and Supplementary Table 2). pH was significantly different between humans, pigs, and controls (ANOVA *p* < 0.05). During the winter, the pH was not as strongly affected: there were no significant changes over the first 5 weeks of the winter trial. After this time, at both the lower and upper sites, the decomposition soils had slightly reduced pH compared to controls; though this was only significant at weeks 5 (229 ADD) and 21 for the lower sites and week 17 (738 ADD) for the upper site (Figures 1B,C and Supplementary Table 3).

**FIGURE 2:**
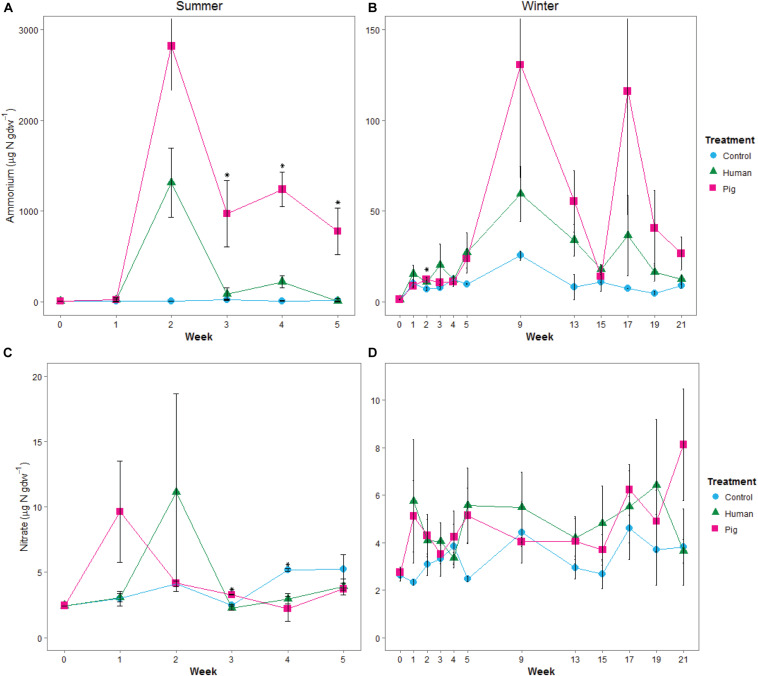
Mean concentrations of ammonium **(A,B)** and nitrate **(C,D)** in soils below decomposing humans and pigs during the summer **(A,C)** and winter **(B,D)** trials. Error bars show standard deviations. Asterisks indicate sample times with significant differences between the three treatments (*p* < 0.05).

Ammonium concentrations were significantly elevated in decomposition soils in both the summer and winter trials (Figures 2A,B). The magnitude of this pulse was much higher in summer, with maximum concentrations reaching >1,000 μgN gdw^–1^ at week 2 (297 ADD); in the winter, peak concentrations were 60 to 125 μgN gdw^–1^ at week 9 (315 ADD). During the summer trial, pigs resulted in a significantly greater pulse of ammonium to the soil compared to controls starting in week 2 and through the remainder of the trial. Human treatments had elevated soil ammonium concentrations, but because of high variability between individuals (Supplementary Table 2), were not significantly different from the control soils in the summer (Figure 2A). In the winter trial, elevated ammonium was observed under decomposing pigs, with maximum concentrations reached at weeks 9 and 17 (315 and 738 ADD) (Figure 2B and Supplementary Table 3). Nitrate concentrations did not significantly change in any of the treatments for either trial (Figures 2C,D and Supplementary Tables 2, 3). Control sites (i.e., background soils) were not significantly different between summer and winter trials for pH, ammonium, or nitrate.

### Soil Microbial Activity

**FIGURE 3:**
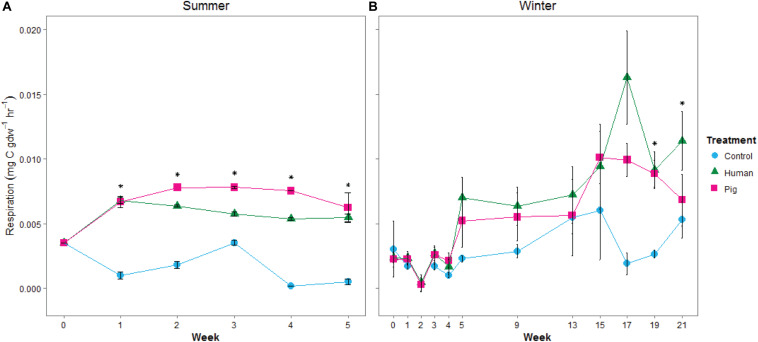
Mean soil respiration rates, estimated from 24-h incubations at 20°C, from soils under decomposing humans and pigs during **(A)** summer and **(B)** winter trials. Error bars show standard deviations. Asterisks indicate significant differences between the three treatments (*p* < 0.05).

Decomposition resulted in elevated soil respiration rates starting in the first week of the summer trial and continuing throughout the experiment. Both human and pig treatments had significantly higher soil respiration rates compared to control soils, and pig-decomposition soils were significantly higher than human-decomposition soils in the summer (Figure 3A and Supplementary Table 2). In the winter trial, elevated respiration rates started during the fifth week (230 ADD), and peaked at weeks 17 through 21 following a period of spring warming (Figure 3B and Supplementary Table 3). Despite differences in ambient temperatures, the magnitude of the increases in soil respiration rates were comparable between summer and winter trials. We also noted that respiration rates were strongly correlated to TBS scores reported by Dautartas et al. (2018): In the summer trial, Pearson’s *r* = 0.54, 0.85 for humans and pigs, respectively; in the winter, *r* = 0.85, 0.84 for humans and pigs, respectively.

**FIGURE 4:**
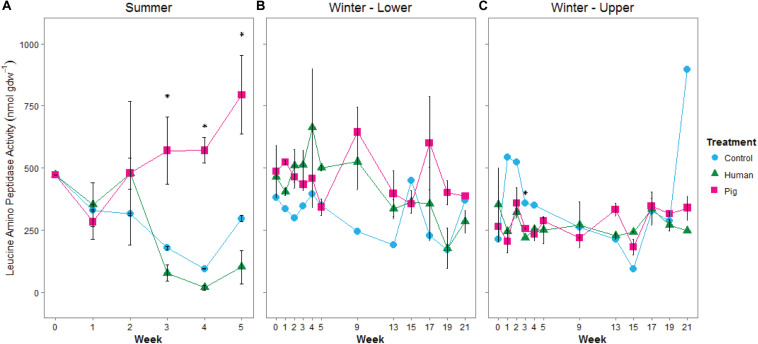
Mean leucine aminopeptidase potential activity in soils below decomposing humans and pigs during summer **(A)** and winter **(B,C)** trials. The lower site **(B)** refers to undisturbed forest soil, while the upper site **(C)** had disturbed soil. Error bars show standard deviations. Asterisks indicated significant differences between the three treatments (*p* < 0.05).

We additionally measured leucine aminopeptidase (LAP) activity as a proxy for potential protease (i.e., protein degradation) capacity of the soil communities. During the summer trial, we observed elevated LAP potential activity in soils below pigs, but not below humans (Figure 4A and Supplementary Table 2). LAP activity was significantly correlated to pH (Spearman rank correlation coefficient *R*_s_ = 0.739, *p* < 0.001, Supplementary Figure 3). During the winter trial, enzyme rates were highly variable. Interestingly, during the first half of the experiment, LAP rates were elevated as a result of decomposition on the organic matter-rich soil of the lower site, but decreased as a result of decomposition in the disturbed soil at the upper site (Figures 4B,C and Supplementary Table 3). Control sites (i.e., background soils) were not significantly different between summer and winter trials for respiration or LAP.

### Metabolomics and Lipidomics (Winter Trial)

**FIGURE 5:**
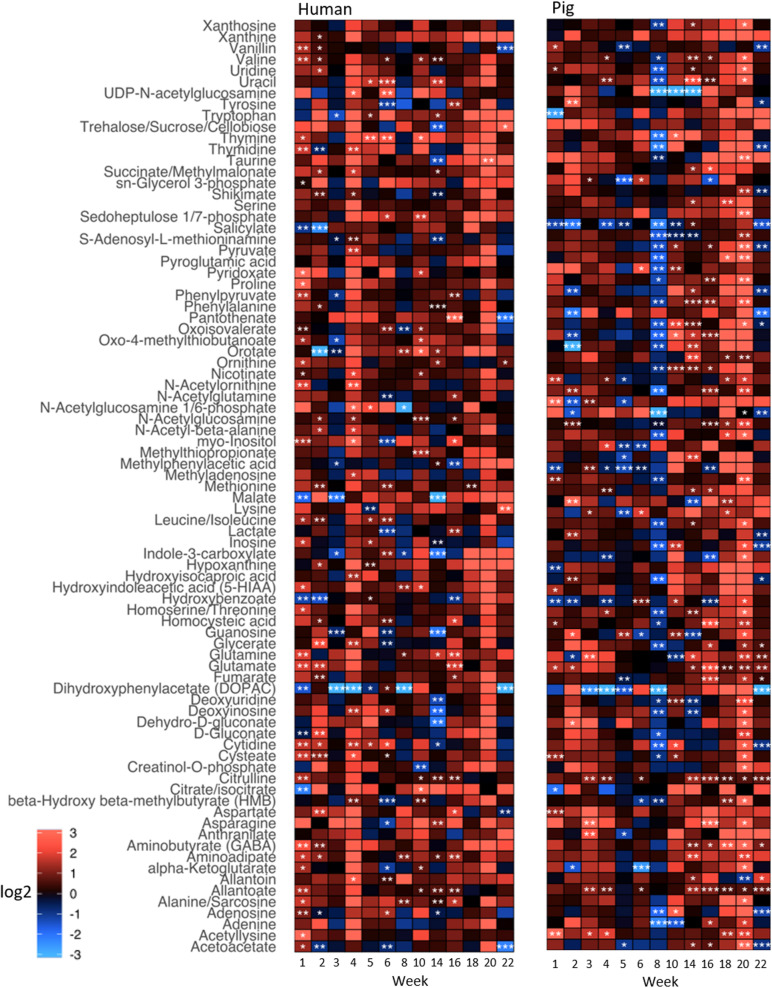
Heatmap depicting relative intensities of metabolites detected in soils below decomposing humans and pigs relative to control soils for the winter trial. Shades of red and blue show an increase and decrease of metabolite intensities, respectively, relative to the control (i.e., fold change). The color scale represents the magnitude of a log2-fold change. Different significance levels (Student’s *t*-test) are indicated with asterisks: *p ≤ 0.1, **p ≤ 0.05, ***p ≤ 0.01.

**FIGURE 6:**
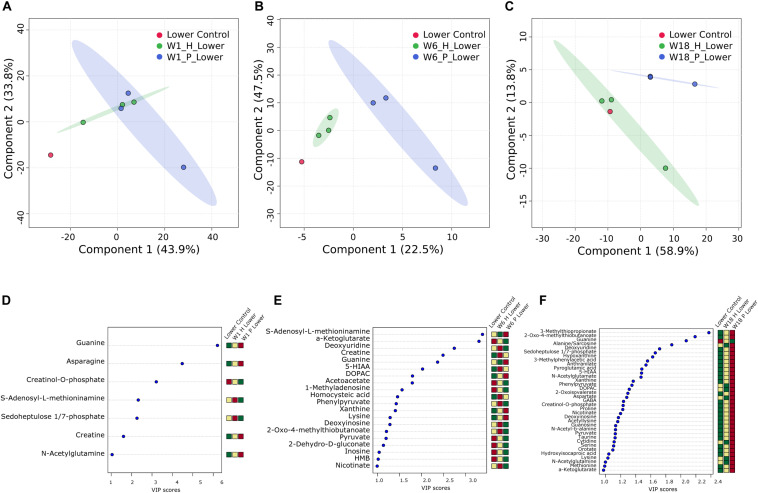
Multivariate analysis of decomposition and control soil from the lower site for week 1 **(A,D)**, week 6 **(B,E)**, and week 18 **(C,F)** for the winter trial. PLS-DA plots **(A–C)** and VIP score plots **(D–F)** for metabolites with VIP > 1 are shown. Heatmaps indicate high (red) and low (green) relative intensity of particular metabolites in human (H) and pig (P) decomposition soil in comparison to control soil.

**FIGURE 7:**
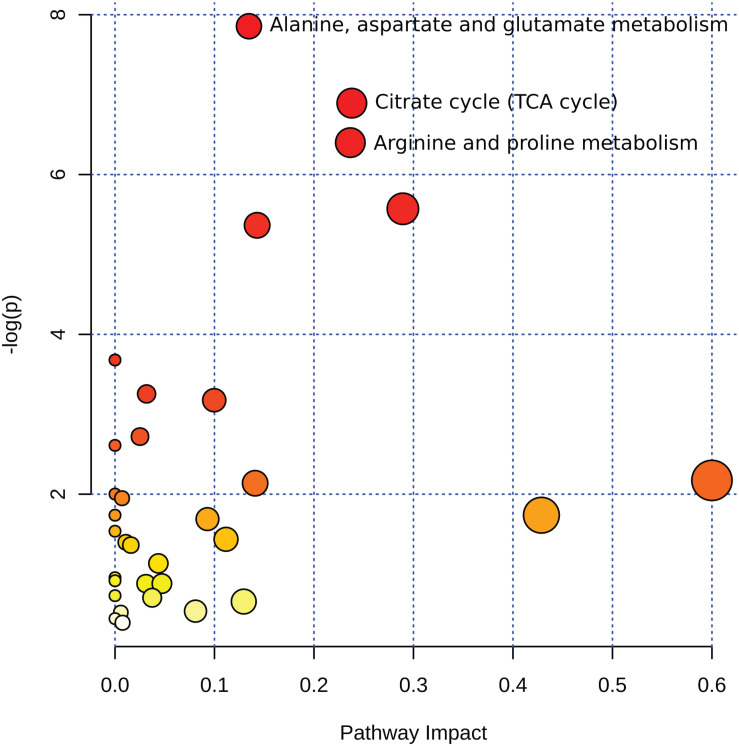
Summary of pathway analysis from 38 metabolites detected in decomposition soil for the winter trial. Circles represent matched pathways according to *p*-values from the pathway enrichment analysis and pathway impact values from the pathway topology analysis. The *x*-axis shows the pathway impact value computed from the pathway topological analysis, also represented by the size of the circle, with larger circles indicating greater pathway impact value. The *y*-axis shows the log of the *p*-values obtained from pathway enrichment analysis, also represented in color, with darker colors indicating lower *p*-values. Alanine, aspartate, and glutamate metabolism: *p* < 0.001, impact value = 0.13462; TCA cycle *p* < 0.001, impact value = 0.23804; arginine and proline metabolism: *p* < 0.001, impact value = 0.23652.

To explore the impact of decomposition on microbial community function and decomposition products, metabolite and lipid profiles were generated for the winter trial soil samples (summer trial samples were not stored at −80°C and therefore could not be used for metabolomics). As with pH and LAP, PLS-DA analysis for metabolomics and lipidomics showed significantly different metabolic profiles in decomposition soil from the upper and lower sites (Supplementary Figure 4), thus we analyzed the two locations separately. Metabolomics analysis revealed a total of 84 metabolites identified from human and pig decomposition soils (Figure 5). PLS-DA of metabolite profiles at each sample timepoint showed significant changes in decomposition soils compared to control soils (Figures 6A–C and Supplementary Figures 5, 6). Individual metabolites responsible for driving the differences in the PLS-DA were identified by variable importance in projection (VIP) scores: Metabolites with VIP scores greater than 1 were identified for each timepoint and used for further analyses. Based on VIP scores, each metabolite was categorized as being high or low in abundance in the three treatments (human, pig, and control) (Figures 6D–F). At the lower site (undisturbed soil), 66 metabolites with VIP > 1 increased in the decomposition treatments compared to control soils, with anthranilate, creatine, 5-hydroxyindoleacetic acid 5 (HIAA), taurine, and xanthine being the most frequently detected (detected in at least 5 out of 13 weeks). In the upper disturbed sites, 48 metabolites with VIP > 1 were identified. Here, *N*-acetylglutamine, acetyllysine, creatine, HIAA, and sedoheptulose 1/7-phosphate were the most frequently detected (detected in at least 4 out of 13 weeks) (Supplementary Figure 7). Combining the two sites, we found a total of 38 metabolites elevated in decomposition soil (Supplementary Table 4). We further assessed those metabolites by matching them to KEGG pathways using the pathway enrichment tool in MetaboAnalyst. Decomposition soils were significantly enriched in metabolites belonging to metabolic pathways of amino acids (e.g., alanine, aspartate, and glutamate, as well as arginine and proline metabolism), and the citric acid (TCA) cycle (Figure 7). We detected multiple amino acids in decomposition soils, such as alanine, aspartate, gamma aminobutyric acid (GABA), glutamate, glutamine, isoleucine, leucine, methionine, phenylalanine, proline, serine, threonine, tyrosine, and valine (Figure 5).

A similar approach was taken for analyzing the lipidomics data via multivariate analysis. The focus was directed to six lipid classes, namely phosphatidylglycerol (PG), phosphatidylethanolamine (PE), phosphatidylinositol (PI), phosphatidic acid (PA), and phosphatidylserine (PS), all known to be major components of bacterial membranes, as well as monogalactosyldiacylglycerol (MGDG), a lipid class present in plants with roles in photosynthesis. Soil from the lower site revealed a total of 84 lipids, whereas the upper location had 57. In detail, we were able to identify 16 PS lipids, 9 PI, 5 PG, 16 PE, 12 PA, and 26 MGDG for the lower site. A similar trend was noticed for the upper sampling location with 11 PS, 7 PI, 3 PG, 14 PE, 5 PA, and 17 MGDG. Combining both results, 54 lipids were elevated in decomposition soils compared to the control soils (Supplementary Table 5).

**FIGURE 8:**
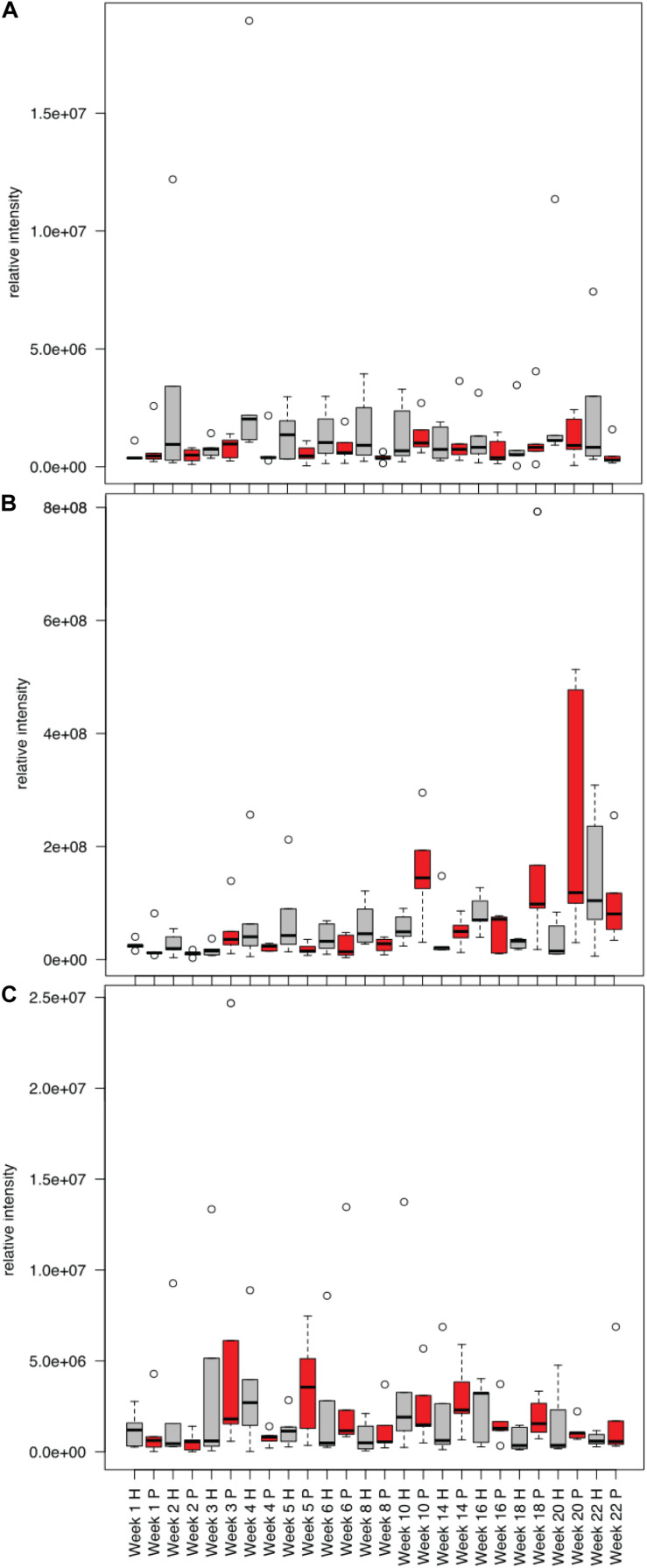
Mean relative intensities of metabolites detected in decomposition soil that were elevated in humans (H, gray) compared to pigs (P, red) for the winter trials: **(A)** sn-glycerol 3-phosphate, **(B)** 2-Oxo-4-methyladenosine, **(C)** tryptophan.

Comparing the human and pig treatments, we saw that by the second week of decomposition, the metabolite profiles in the soils below human and pigs significantly diverged, and these differences continued through the end of the 22-week experiment (Supplementary Figures 5, 6). To determine if there was a difference between human and pig decomposition products, we identified features that were differentially abundant between human and pig plots. Metabolites that were frequently elevated in human decomposition soils compared to pig soils included 2-oxo-4-methylthiobutanoate, sn-glycerol 3-phosphate and tryptophan; these metabolites were elevated at four or more of our sampling time points (Figure 8).

## Discussion

### Signatures of Decomposition-Impacted Soils

Soils beneath decomposing pigs and humans were impacted in terms of the chemical parameters measured in our study. In contrast to controls, which were not exposed to decomposition, we observed significant changes in pH, nutrients, microbial activity and metabolites. The pH change was variable in response and differed between seasons, with pH being affected more drastically in the summer. It was also notable that the pH change was opposite for pigs and humans; pH increased under pigs, but decreased under humans. This variability in soil pH is important to note because soil pH has been determined to be a predictor of bacterial community structure (Lauber et al., 2009), and thus would ultimately influence the composition of the bacterial decomposer communities. Other studies examining the impact of animal or human decomposition on soils have also reported mixed results with respect to pH. For example, depending on the study, decomposing humans on soil surfaces resulted in decreased pH (Aitkenhead-Peterson et al., 2012) or no significant change in pH (Cobaugh et al., 2015; Fancher et al., 2017). Decomposing pigs have been reported to increase soil pH (Benninger et al., 2008; Meyer et al., 2013; Szelecz et al., 2018). Decomposing rabbits also resulted in an increase in pH (Quaggiotto et al., 2019). It has also been noted that buried carcasses (both animal and human) generally cause an increase in pH (Hopkins et al., 2000; Wilson et al., 2007; Stokes et al., 2013; Keenan et al., 2018a). Our study has added to the growing body of observations that the response of soil pH to mammalian decomposition is not predictable and seems to depend on species and local environmental and edaphic conditions.

In the decomposition-impacted soils, we observed an expected increase in soil respiration as an indicator of increased microbial metabolism, which correlated with previously published Total Body Score data, a scale used to score extent of visible morphological decomposition (Dautartas et al., 2018). Many of the metabolites enriched in decomposition soils were intermediates of the TCA cycle, the central energy-generating cycle of aerobic organisms, indicating increased aerobic metabolism. We also observed multiple lines of evidence of protein decomposition and ammonification, including increased protease activity and products of proteolysis, including amino acids and ammonium. We were able to detect several amino acids in the decomposition soils, and pathway analysis showed that many of the enriched metabolites in decomposition soils belonged to amino acid pathways. Our observation is consistent with other studies that have detected amino acids in porcine decomposition fluids (Swann et al., 2012), and soils impacted by human and other mammalian decomposition (Vass et al., 2002; Macdonald et al., 2014). In our study, the most consistently detected amino acids in decomposition soils (both human and pig) were creatine, alanine, proline, taurine and GABA. Creatine is a non-protein amino acid involved in ATP generation in muscle tissue. Studies with rats and mice have shown marked increases in creatine in blood and tissues over the early (24–72 h) postmortem interval (Dai et al., 2019; Mora-Ortiz et al., 2019). Taurine is particularly abundant in bile and the large intestines of mammals. Alanine and proline are two of the more common amino acids that make up proteins in vertebrates and have been detected in the serum, blood and tissues of mice and rats in the first 72 h postmortem (Sato et al., 2015; Kaszynski et al., 2016; Dai et al., 2019; Mora-Ortiz et al., 2019). While human cell autolytic processes would be responsible for the production of amino acids and ammonium early in decomposition, the elevated microbial respiration and protease activities measured in our soils indicated microbially-mediated decomposition was occurring as well.

The proteolytic conversion of proteins into amino acids and ammonium (via both autolytic and microbial-mediated processes) is what ultimately contributes to the pulse of nitrogen introduced to the environment and available to microbes and plants (Macdonald et al., 2014). Ammonium can then be converted to nitrate by nitrification (mediated by nitrifying bacteria and/or archaea); however, only minimal changes in nitrate concentrations were observed here. Elevated ammonium concentrations without a concurrent increase in nitrate concentrations has been consistently observed during the mass loss period of both human and animal carcass decomposition (Macdonald et al., 2014; Cobaugh et al., 2015; Keenan et al., 2018a, b). Longer term studies have noted increases in soil nitrate during skeletonization, once soil oxygen levels have returned and ammonia concentrations have declined (Metcalf et al., 2016; Keenan et al., 2018b; Szelecz et al., 2018), however, our study was not long enough in duration to observe this. Nitrification is an oxygen-dependent process, and elevated respiration in decomposition soils during soft tissue decomposition draws down soil oxygen, limiting nitrification (Geets et al., 2006; Keenan et al., 2018b). In addition, high concentrations of ammonium can be toxic to nitrifying microbes (Vadivelu et al., 2007). Together, our results confirm that mammalian decomposition initially results in enhanced microbial ammonification and limited nitrification in soil during soft tissue decomposition.

### Seasonal Differences in Decomposition Soils

We observed different responses in soil physicochemistry between the summer and winter trials. Notably, pH and nutrient concentrations were more affected in summer: Ammonium concentrations were an order of magnitude higher and nitrate concentrations were twice as high in the summer compared to winter. Soil and internal temperatures during the summer study were elevated during the early stages of decomposition, as has been documented in other studies (Keenan et al., 2018b; Quaggiotto et al., 2019), concurrent with the period of peak fly larvae activity. Larval masses have been shown to generate heat in excess of 10°C above ambient temperatures (Weatherbee et al., 2017). Because of warmer temperatures, increased insect activity, and reduced scavenging, decomposition progressed more rapidly in the summer (Dautartas et al., 2018; Steadman et al., 2018). In the winter, lower ambient temperatures and little to no insect activity (Dautartas et al., 2018) resulted in a slower release of decomposition products over time. This would have given the soil microbial populations more time to assimilate and oxidize the available ammonium. Interestingly, despite differences in ambient temperatures and decomposition rates between summer and winter, levels of microbial activity (i.e., respiration) were comparable between the two seasons.

The elevated microbial activities corresponded to increased soil temperatures in both trials. It was also notable that despite relatively similar microbial respiration rates, we had higher protease activity in the summer compared to winter. This higher degree of specific protease activity in summer is likely due to the more concentrated pulse of decomposition products in soil over a shorter period of time (i.e., higher substrate concentrations). The large larval masses feeding on the donors in the summer may also have contributed to elevated protease activities, as these larvae are known to secrete proteases (Pinilla et al., 2013). Other decomposition studies have reported seasonal differences in decomposition processes, for example, different microbial community compositions (Breton et al., 2016), number and abundance of volatile organic compounds produced (Forbes et al., 2014) and differential transformation of fatty acids in decomposition fluid (Ueland et al., 2018).

### Comparison of Human and Pig Decomposition Soils

The soil biogeochemical responses measured in our study were not the same between pigs and human. Pig decomposition sites had higher ammonium, pH, and LAP activity compared to humans. In addition, the suite of metabolites measured were significantly different between the two species. Metabolites that were frequently elevated in human decomposition soils compared to pig decomposition soils included: 2-Oxo-4-methyladenosine (a modified tRNA), sn-glycerol 3-phosphate (a component of glycerophospholipids that make up biological membranes) and the amino acid tryptophan. Humans and pigs generally attract similar postmortem insect communities (Matuszewski et al., 2020), so the difference we observed between the two species may be because of more extensive scavenging of humans compared to pigs, which would have diverted more of the resource away from decomposers toward scavengers (Steadman et al., 2018). The differences may also have been due to differences in body composition and/or microbiome between the two species. Pigs tend to have higher moisture content (Carter et al., 2007), which might explain increased microbial activity and mobilization of decomposition products into the soil. Pigs also have higher levels of total saturated fatty acids (Notter et al., 2009) and produce different volatile organic compounds during decomposition (Vass et al., 2008) indicating that different body compositions may influence decomposers and their processes. Both humans and pigs are monogastric omnivores and have similar gut microflora compared to other species (Ley et al., 2008). However, the microbiomes of humans and pigs are not identical, and therefore it could be hypothesized that different microflora, which are active participants in postmortem decomposition, could result in different decomposition rates and pathways/metabolites between the two species.

### Importance of Edaphic Properties in Decomposition Response

An unintentional confounding factor in this experiment was discovered once the winter trial was already underway: Some of the subjects had been placed on undisturbed, O/A-horizon soil forest soil, while others were placed on a section where the soil had been disturbed and B horizon subsoil was present. While soil type and parent material were the same, the two sites had different pH and organic matter content. Thus, this allowed us to determine if the starting soil chemistry had an effect on the decomposition response. During decomposition, pH and protease responses were significantly different between the two sites: Decomposition in the soil organic matter (SOM)-rich acidic topsoil resulted in a slight increase in pH and LAP activity, while decomposition in the SOM-poor disturbed subsoil resulted in a slight decrease in pH and LAP activity. This suggests that some of the effects of decomposition on soils may be dependent on soil chemistry or organic matter, and may be an explanation for the inconsistent effects on soil pH that have been previously reported in the literature (Vass et al., 1992; Towne, 2000; Benninger et al., 2008; Aitkenhead-Peterson et al., 2012; Cobaugh et al., 2015; Fancher et al., 2017). SOM can buffer added acids, preventing pH decreases (Jiang et al., 2018), which may explain why we saw an increase in pH in the SOM-rich site and decrease at the SOM-poor site. We additionally show here that protease activity and metabolite profiles were different at the two sites, indicating that the biological communities were also differentially affected.

### Study Limitations

As with many field taphonomy studies conducted at anthropology research facilities, we were limited in terms of donor numbers (*n* = 5 in each treatment group) and spatial constraints (vegetation and terrain) rendered it difficult to sample some soils. Despite best attempts to standardize the placement locations of the donors and carcasses, it was discovered after the winter trial was underway that because of background environmental heterogeneity, there was a significant block (plot) effect that had to be taken into account in the statistical analyses. Finally, storage of the summer trial samples at a higher temperature (−20°C instead of −80°C) left them unsuitable for metabolomics analyses, so unfortunately, we were not able to make a seasonal comparison of metabolomic and lipidomic profiles.

## Conclusion

A direct comparison of humans and pigs has shown that their decomposition dynamics (Dautartas et al., 2018) and impacts on soils (this study) are not identical. Given the accepted use of animal analogs in forensic taphonomy research, this study further contributes to the growing understanding of limitations with this practice. This work also has implications in ecosystems ecology. Decaying carcasses are an important part of nutrient cycling in ecosystems; differences between species in terms of nutrient redistribution may be important to consider as animal populations change in space and time. Finally, an unintended finding of our study was the differential decomposition response between organic matter rich topsoil and organic matter poor subsoil, demonstrating that microbial and biogeochemical responses may be dependent on local edaphic properties; an aspect which should be investigated in future studies.

## Data Availability Statement

The datasets generated for this study can be found in the Supplementary Material and in online repositories. The names of the repository/repositories and accession number(s) can be found below: MetaboLights Database, (https://www.ebi.ac.uk/metabolights) under ID MTBLS2254.

## Ethics Statement

The studies involving human participants were reviewed and approved by University of Tennessee Institutional Review Board. Written informed consent for participation was not required for this study in accordance with the national legislation and the institutional requirements. The animal study was reviewed and approved by University of Tennessee Institutional Animal Care and Use Committee. Written informed consent was obtained from the owners for the participation of their animals in this study.

## Author Contributions

The study was conceived by JD, DS, AM, and GV. Resources were provided by JD, DS, AM, and SC. Field experiments, sampling, and laboratory analyses were conducted by JD, AD, JS, MM, and SB. Metabolomics and lipidomics laboratory and data analyses were conducted by KH, MM, SD, HC, and KH. Data analysis was conducted by JD, KH, LT, JS, and AD. Manuscript writing was led by JD, KH, and LT with input from all authors.

## Conflict of Interest

The authors declare that the research was conducted in the absence of any commercial or financial relationships that could be construed as a potential conflict of interest.

## Footnotes

1 https://fac.utk.edu/

2 https://www.ebi.ac.uk/metabolights
