# Supplementary material for: Comparative Decomposition of Humans and Pigs: Soil Biogeochemistry, Microbial Activity and Metabolomic Profiles
Source: Front Microbiol. 2021 Jan 13;11:608856. doi: 10.3389/fmicb.2020.608856 (PMC7838218; doi:10.3389/fmicb.2020.608856)
Supplement: Supplementary file 1 [file Data_Sheet_1.docx]

***Supplementary Material***

**Comparative decomposition of humans and pigs: Soil biogeochemistry, microbial activity and metabolomic profiles**

Jennifer M. DeBruyn^1^*, Katharina M. Hoeland^2^, L. Stacy Taylor^1^, Jessica D. Stevens^1^, Michelle A. Moats^1^, Sreejata Bandopadhyay^1#^, Stephen P. Dearth^2^, Hector F. Castro^3^, Kaitlin K. Hewitt^2#^, Shawn R. Campagna^3^, Angela M. Dautartas^3#^, Giovanna M. Vidoli^3^, Amy Z. Mundorff^3^, Dawnie W. Steadman^3^

^1^ Department of Biosystems Engineering & Soil Science, University of Tennessee

^2^ Department of Chemistry, University of Tennessee

^2^ Biological and Small Molecule Mass Spectrometry Core, Department of Chemistry, University of Tennessee

^3^ Department of Anthropology, University of Tennessee

**Number of Supplementary Tables: 5**

**Number of Supplementary Figures: 7**

**Table S1.** Sampling dates for summer and winter trials. Total Body Score (TBS) and Accumulated Degree Days (ADD) are from Dautartas et al. (2018).

| **Week Number** | **Date** | **Total Body Score (TBS)** | | **Accumulated Degree Days (ADD)** |
| --- | --- | --- | --- | --- |
|  |  | Humans | Pigs |  |
| **Summer trial** |  |  |  |  |
| 0 | 2014-06-10 | 3 | 3 | 0.0 |
| 1 | 2014-06-17 | 14 | 15 | 122.5 |
| 2 | 2014-06-24 | 21 | 27 | 297.9 |
| 3 | 2014-07-01 | 25 | 29 | 465.7 |
| 4 | 2014-07-08 | 25 | 29 | 663.7 |
| 5 | 2014-07-15 | 26 | 30 | 815.8 |
| **Winter trial** |  |  |  |  |
| 0 | 2014-12-04 | 3 | 3 | 0.0 |
| 1 | 2014-12-08 | 8 | 6 | 85.3 |
| 2 | 2014-12-15 | 9 | 8 | 101.5 |
| 3 | 2014-12-22 | 10 | 10 | 144.5 |
| 4 | 2014-12-29 | 10 | 10 | 197.7 |
| 5 | 2015-01-05 | 12 | 13 | 230.0 |
| 9 | 2015-02-03 | 16 | 13 | 314.6 |
| 13 | 2015-03-02 | 16 | 14 | 379.8 |
| 15 | 2015-03-17 | 24 | 15 | 543.6 |
| 17 | 2015-03-31 | 25 | 25 | 738.1 |
| 19 | 2015-04-13 | 26 | 27 | 974.5 |
| 21 | 2015-04-27 | NA* | NA | 1222.0 |

*NA = TBS data was not available.

**Reference:**

Dautartas, A., Kenyhercz, M.W., Vidoli, G.M., Meadows Jantz, L., Mundorff, A., and Steadman, D.W. (2018). Differential Decomposition Among Pig, Rabbit, and Human Remains. Journal of Forensic Sciences 63, 1673-1683.

**Table S2.** Mean ± standard deviation of soil biochemistry variables by sampling date and treatment for summer trial. LAP is potential leucine aminopeptidase activity. For each week, differences between treatments were determined by ANOVA, with significant treatment differences (p < 0.05) indicated in bold. Significantly different groups based on a TukeyHSD posthoc test are indicated by capital letters.

| **Week** | **Treatment** | **pH** | **Ammonium**  **(µgN gdw^-1^)** | **Nitrate**  **(µgN gdw^-1^)** | **Respiration (µgC gdw^-1^ h^-1^)** | **LAP (nmol gdw^-1^ h^-1^)** |
| --- | --- | --- | --- | --- | --- | --- |
| 0 | Initial | 6.93 ± 0.31 | 10.21 ± 0.50 | 2.44 ± 0.10 | 3.50 ± 0.40 | 472.6 ± 6.5 |
| 1 | Control^a^ | 6.935 ± 0.32 | 5.02 ± 0.04 | 2.97 ± 0.80 | **0.98 ± 0.42^A^** | 329.0 ± 5.4 |
|  | Human^b^ | 6.78 ±0.46 | 26.60 ± 36.85 | 3.06 ± 0.67 | **6.78 ± 0.46^B^** | 352.2 ± 174.5 |
|  | Pig^c^ | 6.68 ± 0.78 | 21.33 ± 22.34 | 9.63 ± 6.70 | **6.68 ± 0.78^B^** | 282.6 ± 119.2 |
| 2 | Control | **6.77 ± 0.54^AB^** | **8.36 ± 0.36^A^** | 4.12 ± 0.28 | **1.81 ± 0.40^A^** | 313.8 ± 7.3 |
|  | Human | **6.36 ± 0.19^A^** | **1314.53 ± 769.52^AB^** | 11.11 ± 15.11 | **6.36 ± 0.19^B^** | 477.4 ± 127.2 |
|  | Pig | **7.78 ± 0.31^B^** | **2819.77 ± 522.10^B^** | 4.18 ± 1.01 | **7.78 ± 0.40^C^** | 479.3 ± 407.6 |
| 3 | Control | **6.99 ± 0.32^A^** | **22.39 ± 9.69^A^** | 2.46 ± 0.07 | **3.51 ± 0.26^A^** | **177.1 ± 8.7^A^** |
|  | Human | **5.73 ± 0.34^B^** | **87.92 ± 130.33^A^** | 2.26 ± 0.31 | **5.73 ± 0.34^B^** | **76.5 ± 66.4^A^** |
|  | Pig | **7.80 ± 0.13^C^** | **970.69 ± 517.61^B^** | 3.28 ± 0.03 | **7.80 ± 0.13^C^** | **570.2 ± 191.2^B^** |
| 4 | Control | **6.84 ± 0.32^A^** | **11.95 ± 7.76^A^** | 5.16 ± 0.15 | **0.17 ± 0.01^A^** | **93.2 ± 2.4^A^** |
|  | Human | **5.37 ± 0.23^B^** | **221.84 ± 125.50^A^** | 2.96 ± 0.78 | **5.37 ± 0.23^B^** | **17.7 ± 20.8^A^** |
|  | Pig | **7.53 ± 0.01^C^** | **1239.15 ± 267.10^B^** | 2.21 ± 1.37 | **7.53 ± 0.01^C^** | **570.8 ± 73.1^B^** |
| 5 | Control | **7.06 ± 0.28^A^** | **19.62 ± 26.47^A^** | 5.25 ± 1.52 | **0.49 ± 0.28^A^** | **295.9 ± 18.5^A^** |
|  | Human | **5.36 ± 0.48^B^** | **7.29 ± 4.04^A^** | 3.88 ± 1.26 | **5.45 ± 0.55^B^** | **100.6 ± 133.2^A^** |
|  | Pig | **7.36 ± 0.30^C^** | **777.38 ± 360.22^B^** | 3.72 ± 0.10 | **6.23 ± 1.60^B^** | **794.4 ± 224.1^B^** |

^a^n=6, ^b^n=4, ^c^n=3

**Table S3.** Mean ± standard deviation of soil biochemistry variables by sampling date and treatment for winter trial. For each week, differences between treatments were determined by ANOVA, with significant treatment differences (p < 0.05) indicated in bold. Significantly different groups based on a TukeyHSD posthoc test are indicated by capital letters.

| **Week** | **Treatment** | **pH** | **Ammonium (µgN gdw^-1^)** | **Nitrate (µgN gdw^-1^)** | **Respiration (µgC gdw^-1^ h^-1^)** | **LAP (nmol gdw^-1^ h^-1^)** |
| --- | --- | --- | --- | --- | --- | --- |
| 0 | Initial | 7.22 ± 0.30 | 1.30 ± 0.18 | 2.68 ± 0.12 | 2.53 ± 1.15 | 382.2 ± 33.5 |
| 1 | Control^a^ | 7.59 ± 0.81 | 10.04 ± 3.25 | 2.32 ± 0.17 | 1.67 ± 0.26 | 438.6 ± 146.3 |
|  | Human^b^ | 7.54 ± 0.38 | 15.38 ± 11.26 | 5.74 ± 5.82 | 2.32 ± 1.15 | 323.6 ± 93.7 |
|  | Pig^c^ | 7.28 ± 0.37 | 8.83 ± 0.97 | 5.11 ± 3.37 | 2.25 ± 0.82 | 396.0 ± 178.3 |
| 2 | Control | 7.46 ± 0.42 | **7.07 ± 1.45^A^** | 3.07 ± 0.63 | 1.39 ± 0.44 | 410.4 ± 158.0 |
|  | Human | 6.85 ± 0.46 | **10.87 ± 2.20^A^** | 4.08 ± 1.99 | 0.42 ± 1.42 | 415.2 ± 121.3 |
|  | Pig | 7.07 ± 0.28 | **12.56 ± 2.20^B^** | 4.30 ± 1.98 | 0.26 ± 1.25 | 421.3 ± 90.3 |
| 3 | Control | 6.95 ± 0.13 | 7.77 ± 0.89 | 3.31 ± 1.04 | 1.70 ± 0.37 | 352.5 ± 8.7 |
|  | Human | 7.14 ± 0.55 | 20.26 ± 16.30 | 4.04 ± 1.77 | 2.66 ± 1.17 | 413.0 ± 180.4 |
|  | Pig | 7.05 ± 0.38 | 10.64 ± 1.97 | 3.51 ± 0.87 | 2.56 ± 1.58 | 362.2 ± 102.4 |
| 4 | Control | 6.64 ± 1.12 | 12.56 ± 1.66 | 3.85 ± 1.30 | 1.00 ± 0.27 | 373.4 ± 32.2 |
|  | Human | 6.94 ± 0.33 | 12.21 ± 4.58 | 3.33 ± 0.56 | 1.66 ± 1.23 | 457.5 ± 305.7 |
|  | Pig | 7.06 ± 0.52 | 11.15 ± 5.66 | 4.23 ± 2.42 | 2.14 ± 1.30 | 345.1 ± 163.1 |
| 5 | Control | **7.88 ± 0.67^A^** | 9.80 ± 0.18 | 2.47 ± 0.18 | 2.28 ± 0.34 | 350.3 ± 52.2 |
|  | Human | **7.45 ± 0.37^B^** | 27.06 ± 24.87 | 5.57 ± 3.52 | 6.97 ± 3.62 | 375.8 ± 151.4 |
|  | Pig | **7.28 ± 0.37^B^** | 23.67 ± 11.47 | 5.13 ± 2.60 | 5.21 ± 4.62 | 319.9 ± 51.4 |
| 9 | Control | 6.50 ± 0.59 | 25.59 ± 3.56 | 4.43 ± 1.81 | 2.87 ± 0.76 | 251.7 ± 12.4 |
|  | Human | 5.99 ± 0.37 | 59.49 ± 33.78 | 5.48 ± 3.30 | 6.33 ± 3.29 | 423.0 ± 207.2 |
|  | Pig | 6.21 ± 0.57 | 130.67 ± 133.43 | 4.03 ± 0.44 | 5.50 ± 4.17 | 474.3 ± 264.6 |
| 13 | Control | 6.44 ± 0.64 | 8.19 ± 10.07 | 2.94 ± 0.68 | 5.45 ± 4.18 | 201.5 ± 17.4 |
|  | Human | 6.85 ± 0.52 | 34.02 ± 19.46 | 4.19 ± 2.01 | 7.21 ± 4.90 | 290.7 ± 76.1 |
|  | Pig | 6.88 ±0.43 | 55.45 ± 37.91 | 4.05 ± 2.14 | 5.64 ± 3.19 | 370.9 ± 119.8 |
| 15 | Control | **7.66 ± 1.56^A^** | 11.06 ± 7.26 | 2.67 ± 0.89 | 6.03 ± 5.45 | 270.8 ± 253.9 |
|  | Human | **6.33 ± 0.81^B^** | 18.00 ± 5.90 | 4.80 ± 3.53 | 9.40 ±7.37 | 314.2 ± 87.1 |
|  | Pig | **6.99 ± 0.93^C^** | 13.93 ± 6.95 | 3.69 ± 1.45 | 10.12 ± 4.52 | 285.6 ± 98.8 |
| 17 | Control | 6.60 ± 0.28 | 7.35 ± 0.07 | 4.61 ± 1.89 | 1.90 ± 1.19 | 277.5 ± 71.0 |
|  | Human | 6.24 ± 0.49 | 36.52 ± 49.67 | 5.49 ± 3.40 | 6.28 ± 8.09 | 343.9 ± 133.7 |
|  | Pig | 6.28 ± 0.28 | 116.10 ± 154.30 | 6.22 ± 2.37 | 9.91 ± 2.88 | 498.6 ± 268.8 |
| 19 | Control | 6.96 ± 0.75 | 4.80 ± 1.31 | 3.70 ± 2.10 | **2.64 ± 0.45^A^** | 228.0 ± 83.8 |
|  | Human | 6.67 ± 0.58 | 16.22 ± 11.13 | 6.41 ± 6.20 | **9.13 ± 3.18^B^** | 231.9 ± 81.9 |
|  | Pig | 6.73 ± 0.58 | 40.72 ± 46.28 | 4.90 ± 2.90 | **8.85 ± 2.32^B^** | 365.9 ± 75.3 |
| 21 | Control | **6.92 ± 0.57^A*^** | 8.98 ± 2.20 | 3.82 ± 2.26 | **5.30 ± 1.97^A^** | 633.8 ± 372.7 |
|  | Human | **5.96 ± 1.19^B*^** | 12.33 ±5.39 | 3.64 ± 1.14 | **11.38 ± 5.07^B^** | 270.7 ± 50.2 |
|  | Pig | **6.56 ± 0.45^A*^** | 26.62 ± 20.37 | 8.12 ± 5.24 | **6.82 ± 4.46^B^** | 354.7 ± 55.4 |

^a^n=6, ^b^n=5, ^c^n=5 ; *Significant differences observed at lower site only.

**Table S4:** Metabolites (38) which had increased relative intensities in decomposition soil from humans and pigs compared to control soil.

| Metabolite name | Molecular weight  (g mol^-1^) | Mean relative intensities | | Number of study weeks elevated (VIP>1) | |
| --- | --- | --- | --- | --- | --- |
|  |  | **Decomposition soils** | **Control soils** | **Lower site** | **Upper site** |
| 2-Aminoadipate | 161.16 | 4.16E+06 | 2.25E+06 | 4 | 1 |
| 2-Dehydro-D-gluconate | 194.14 | 1.20E+07 | 7.17E+06 | 1 | 1 |
| 2-Oxo-4-methylthiobutanoate | 148.18 | 6.70E+07 | 3.67E+07 | 4 | 2 |
| 2-Oxoisovalerate | 116.11 | 1.96E+08 | 1.35E+08 | 3 | 1 |
| 3-Methylthiopropionate | 120.17 | 3.39E+06 | 2.81E+06 | 4 | 3 |
| 4-Aminobutyrate (GABA) | 103.12 | 2.53E+06 | 1.83E+06 | 2 | 3 |
| 5-Hydroxyindoleacetic acid (5-HIAA) | 191.18 | 3.14E+05 | 7.79E+04 | 5 | 4 |
| Acetoacetate | 102.09 | 9.77E+07 | 9.41E+07 | 1 | 2 |
| Acetyllysine | 188.22 | 7.67E+05 | 3.76E+05 | 2 | 5 |
| Alanine/Sarcosine | 89.09 | 1.08E+07 | 6.85E+06 | 2 | 3 |
| Allantoate | 176.13 | 5.79E+05 | 4.51E+05 | 2 | 1 |
| alpha-Ketoglutarate | 146.10 | 7.61E+07 | 5.06E+07 | 2 | 2 |
| Anthranilate | 137.14 | 1.51E+05 | 1.36E+05 | 5 | 2 |
| Asparagine | 132.12 | 8.48E+04 | 3.82E+04 | 4 | 2 |
| Citrate/Isocitrate | 192.12 | 5.51E+05 | 4.48E+05 | 3 | 2 |
| Citrulline | 175.19 | 6.46E+06 | 4.59E+06 | 1 | 1 |
| Creatine | 131.13 | 6.57E+04 | 2.46E+03 | 7 | 4 |
| Creatinol-O-phosphate | 197.13 | 2.40E+05 | 1.43E+05 | 3 | 1 |
| D-Gluconate | 196.15 | 2.61E+07 | 1.82E+07 | 3 | 1 |
| Guanine | 151.13 | 1.75E+05 | 1.29E+05 | 3 | 2 |
| Homocysteic acid | 183.18 | 5.40E+05 | 3.77E+05 | 2 | 2 |
| Indole-3-carboxylate | 161.16 | 1.47E+06 | 1.30E+06 | 2 | 2 |
| Lysine | 146.19 | 1.16E+05 | 6.08E+04 | 2 | 3 |
| Malate | 134.09 | 1.60E+06 | 1.35E+06 | 3 | 3 |
| myo-Inositol | 180.16 | 2.16E+06 | 1.84E+06 | 2 | 1 |
| N-Acetylglutamine | 188.18 | 3.17E+05 | 1.84E+05 | 2 | 4 |
| Ornithine | 132.16 | 2.34E+05 | 1.70E+05 | 2 | 2 |
| Proline | 115.13 | 7.56E+06 | 4.58E+06 | 2 | 3 |
| Pyroglutamic acid | 129.11 | 1.15E+09 | 6.23E+08 | 2 | 2 |
| S-Adenosyl-L-methioninamine | 355.43 | 1.42E+05 | 1.40E+05 | 2 | 1 |
| Sedoheptulose 1/7-phosphate | 290.16 | 1.47E+05 | 9.49E+04 | 3 | 4 |
| sn-Glycerol 3-phosphate | 172.07 | 1.35E+06 | 1.04E+06 | 1 | 1 |
| Taurine | 125.15 | 2.86E+06 | 1.59E+06 | 5 | 2 |
| Thymine | 126.11 | 1.04E+07 | 7.93E+06 | 3 | 2 |
| UDP-N-acetylglucosamine | 607.35 | 4.95E+05 | 4.05E+05 | 1 | 2 |
| Uracil | 112.09 | 5.49E+07 | 3.79E+07 | 1 | 1 |
| Xanthine | 152.11 | 1.24E+07 | 8.20E+06 | 6 | 1 |
| Xanthosine | 284.23 | 6.79E+05 | 5.99E+05 | 2 | 3 |

**Table S5:** Lipid classes (54) which had increased intensities in decomposition soil from humans and pigs compared to control soils.

| Lipid Class | Lipid | Mean relative intensities | | Number of study weeks elevated (VIP>1) | |
| --- | --- | --- | --- | --- | --- |
|  |  | **Decomposition soils** | **Control soils** | **Lower site** | **Upper site** |
| Phosphatidic acid (PA) | PA (40:4) | 2.20E+05 | 1.31E+05 | 2 | 3 |
|  | PA (40:5) | 2.37E+04 | 1.35E+04 | 1 | 2 |
|  | PA (42:4) | 5.07E+05 | 2.68E+05 | 3 | 1 |
|  | PA (42:5) | 5.83E+05 | 3.69E+05 | 3 | 1 |
|  | PA (44:7) | 1.49E+05 | 1.07E+05 | 2 | 1 |
| Phosphatidylglycerol (PG) | PG (32:0) | 4.35E+05 | 2.37E+05 | 5 | 1 |
|  | PG (40:7) | 3.69E+04 | 1.46E+04 | 1 | 1 |
| Phosphatidylethanolamine (PE) | PE (28:0) | 7.44E+04 | 2.40E+04 | 4 | 3 |
|  | PE (32:0) | 3.38E+05 | 1.82E+05 | 3 | 2 |
|  | PE (32:2) | 8.84E+05 | 4.35E+05 | 3 | 1 |
|  | PE (34:0) | 2.30E+05 | 1.16E+05 | 4 | 1 |
|  | PE (34:1) | 4.82E+05 | 2.54E+05 | 3 | 1 |
|  | PE (34:2) | 1.66E+05 | 9.29E+04 | 3 | 2 |
|  | PE (34:3) | 1.04E+05 | 5.40E+04 | 3 | 1 |
|  | PE (36:2) | 1.16E+06 | 6.83E+05 | 3 | 1 |
|  | PE (36:3) | 1.77E+05 | 1.15E+05 | 3 | 1 |
|  | PE (36:4) | 1.02E+05 | 8.25E+04 | 3 | 1 |
|  | PE (38:2) | 1.11E+05 | 5.26E+04 | 2 | 1 |
|  | PE (38:5) | 7.44E+04 | 7.30E+04 | 3 | 1 |
|  | PE (38:6) | 6.27E+04 | 6.02E+04 | 2 | 1 |
|  | PE (44:4) | 4.83E+04 | 3.43E+04 | 2 | 1 |
| Monogalacto-syldiacylglycerol (MGDG) | MGDG (28:0) | 7.36E+04 | 4.90E+04 | 3 | 2 |
|  | MGDG (28:6) | 2.62E+04 | 1.93E+03 | 3 | 1 |
|  | MGDG (30:0) | 3.68E+05 | 2.76E+05 | 2 | 1 |
|  | MGDG (30:2) | 7.49E+04 | 6.00E+04 | 3 | 2 |
|  | MGDG (32:0) | 3.54E+05 | 2.61E+05 | 3 | 2 |
|  | MGDG (32:1) | 4.37E+05 | 2.53E+05 | 2 | 2 |
|  | MGDG (32:2) | 2.04E+05 | 1.27E+05 | 3 | 2 |
|  | MGDG (32:4) | 1.05E+05 | 9.00E+04 | 2 | 1 |
|  | MGDG (34:3) | 2.34E+05 | 1.50E+05 | 3 | 1 |
|  | MGDG (36:3) | 4.73E+05 | 2.69E+05 | 1 | 1 |
|  | MGDG (38:2) | 1.01E+06 | 4.34E+05 | 3 | 1 |
|  | MGDG (38:5) | 1.72E+05 | 1.22E+05 | 2 | 1 |
|  | MGDG (40:2) | 5.48E+04 | 2.65E+04 | 2 | 1 |
|  | MGDG (40:4) | 3.53E+06 | 1.87E+06 | 3 | 1 |
|  | MGDG (40:6) | 5.16E+05 | 3.49E+05 | 2 | 2 |
|  | MGDG (42:5) | 5.54E+04 | 3.46E+04 | 3 | 1 |
| Phosphatidylinositol (PI) | PI (32:1) | 8.10E+04 | 5.63E+04 | 3 | 1 |
|  | PI (34:2) | 1.53E+05 | 1.42E+05 | 4 | 1 |
|  | PI (34:0) | 1.16E+05 | 6.74E+04 | 3 | 1 |
|  | PI (36:0) | 3.65E+04 | 1.89E+04 | 1 | 1 |
|  | PI (34:1) | 2.04E+05 | 1.06E+05 | 4 | 1 |
|  | PI (36:1) | 3.02E+04 | 1.54E+04 | 2 | 1 |
|  | PI (36:3) | 4.05E+04 | 3.47E+04 | 2 | 1 |
| Phosphatidylserine (PS) | PS (34:0) | 4.36E+05 | 2.46E+05 | 3 | 1 |
|  | PS (36:0) | 2.98E+06 | 1.42E+06 | 3 | 2 |
|  | PS (40:1) | 4.72E+06 | 2.40E+06 | 3 | 1 |
|  | PS (42:6) | 3.64E+04 | 2.07E+04 | 2 | 2 |
|  | PS (34:4) | 3.66E+04 | 1.63E+04 | 1 | 1 |
|  | PS (36:2) | 7.51E+04 | 1.15E+05 | 2 | 1 |
|  | PS (40:6) | 7.28E+04 | 6.88E+04 | 3 | 1 |
|  | PS (34:2) | 1.19E+05 | 8.60E+04 | 4 | 1 |
|  | PS (36:1) | 6.62E+05 | 3.78E+05 | 2 | 2 |
|  | PS (40:2) | 1.43E+05 | 7.35E+04 | 2 | 2 |


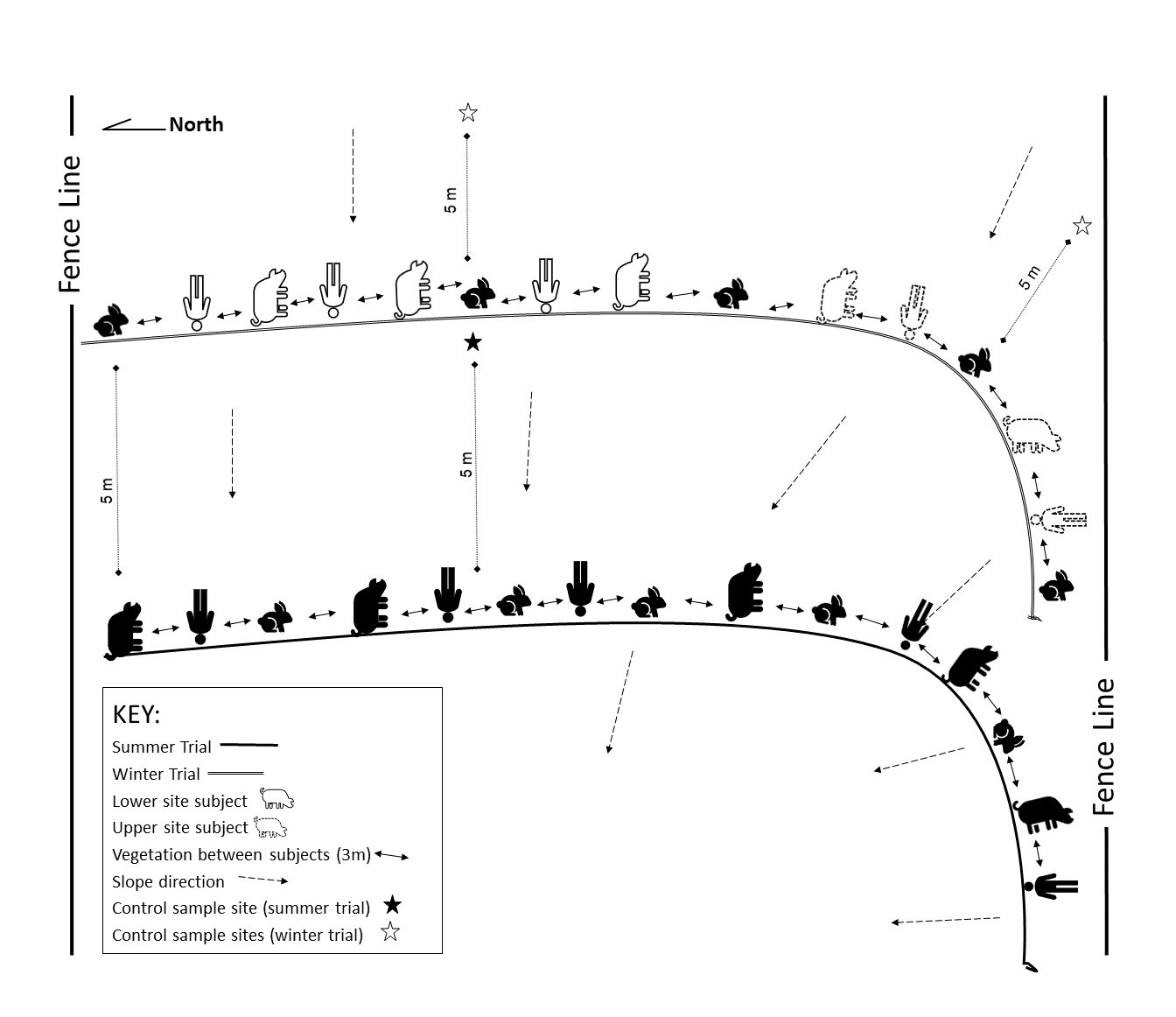


**Figure S1.** Schematic of experimental design layout at the Anthropology Research Facility for summer (closed symbols) and winter (open symbols) trials. Not to scale.


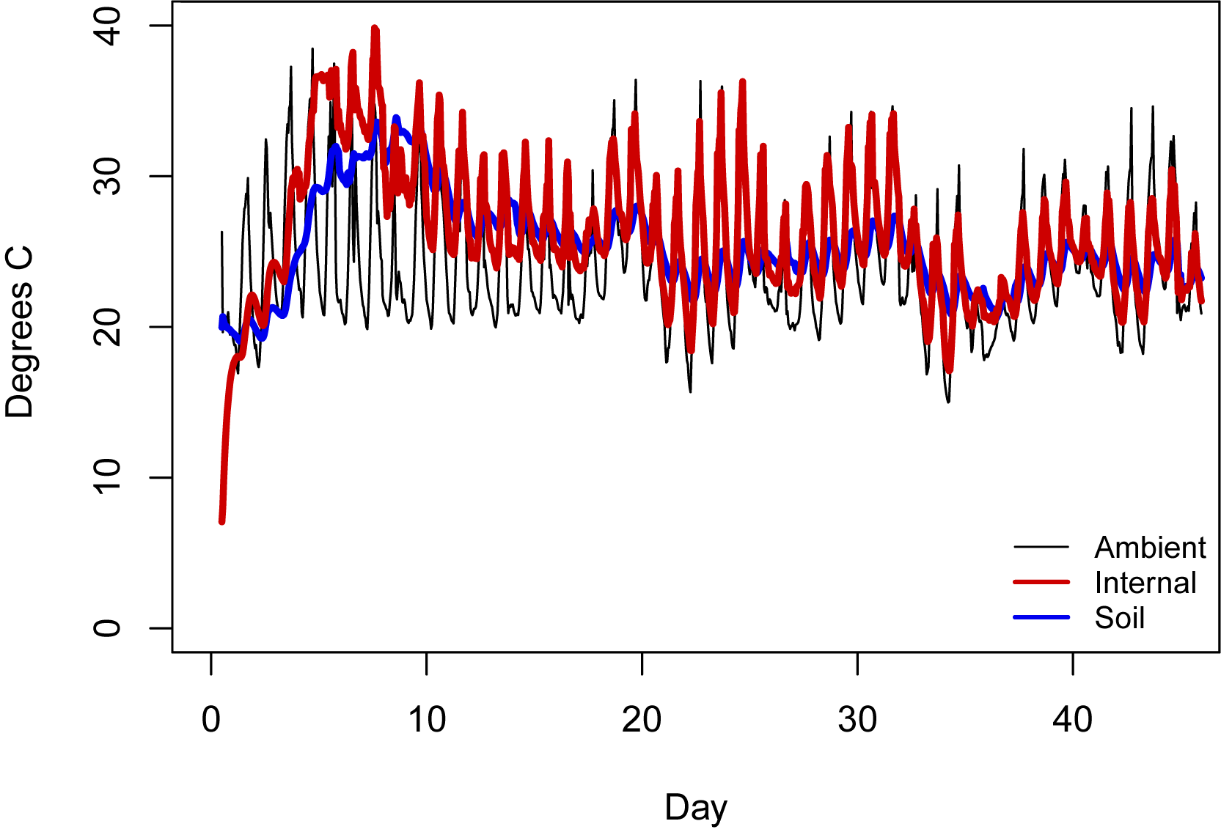


A

B

**
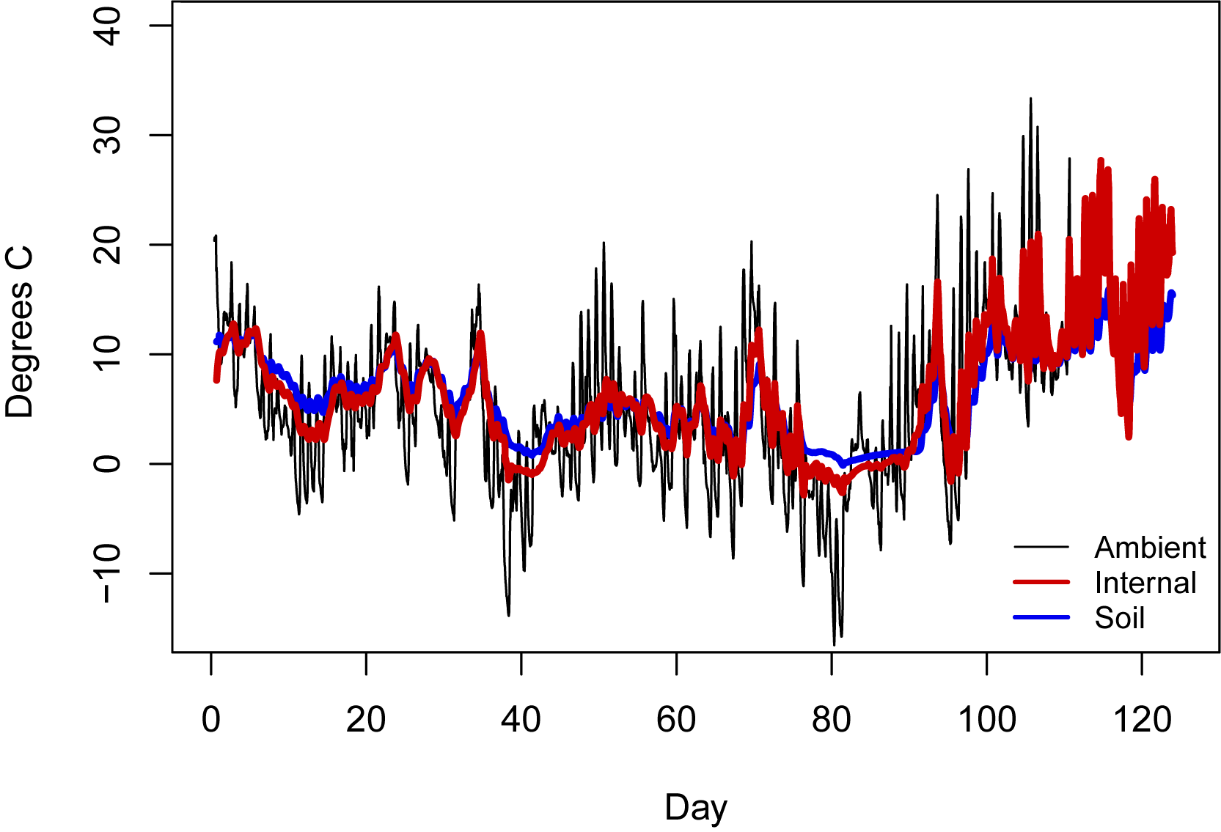
**

**Figure S2.** Mean ambient air (black), internal human donor (red), and soil (blue) temperatures during human decomposition summer (A) and winter (B) trials.


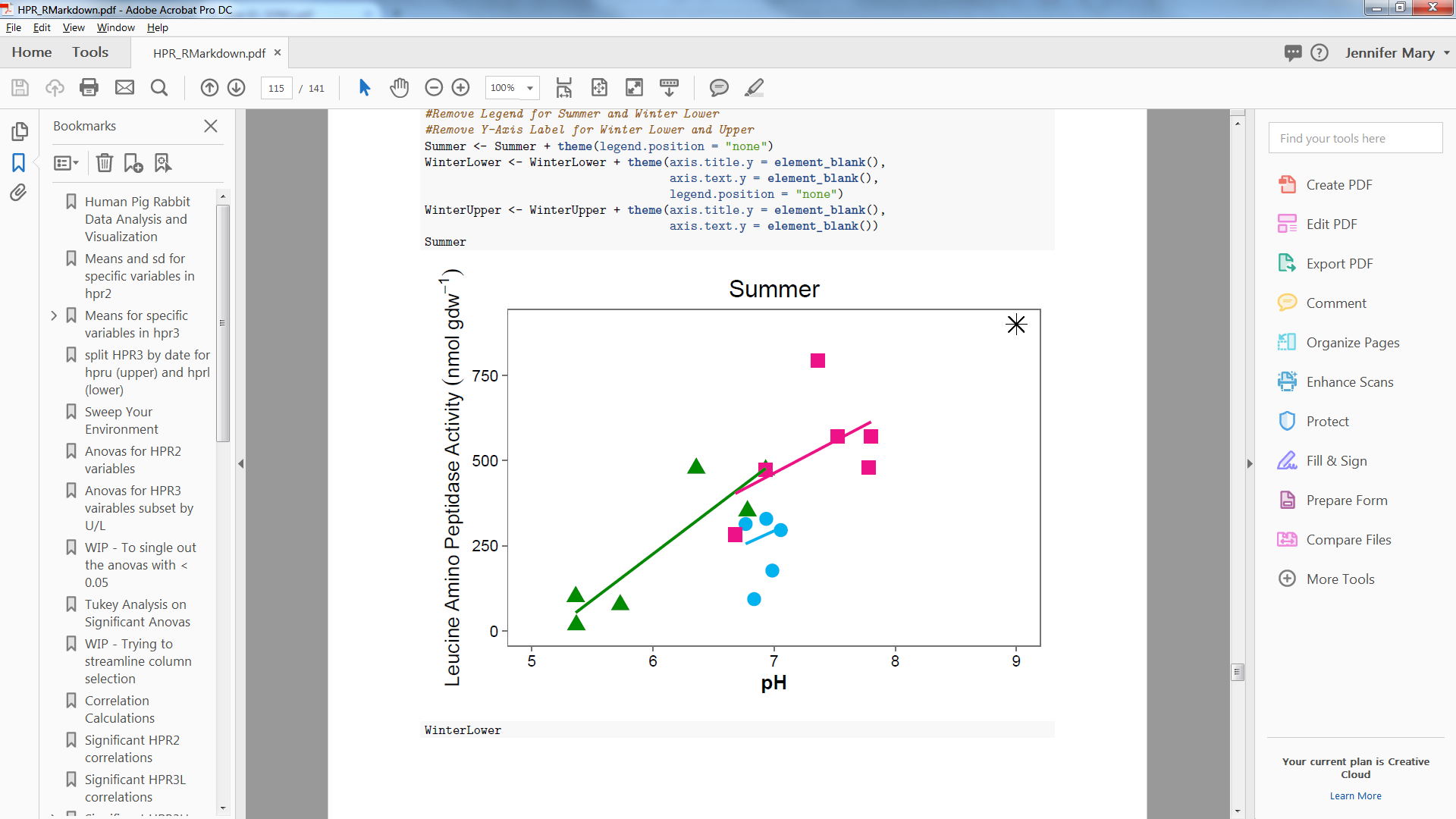


**Figure S3.** Relationship between pH and LAP activity during the summer trial under humans (green), pigs (pink) and at the control site (blue).


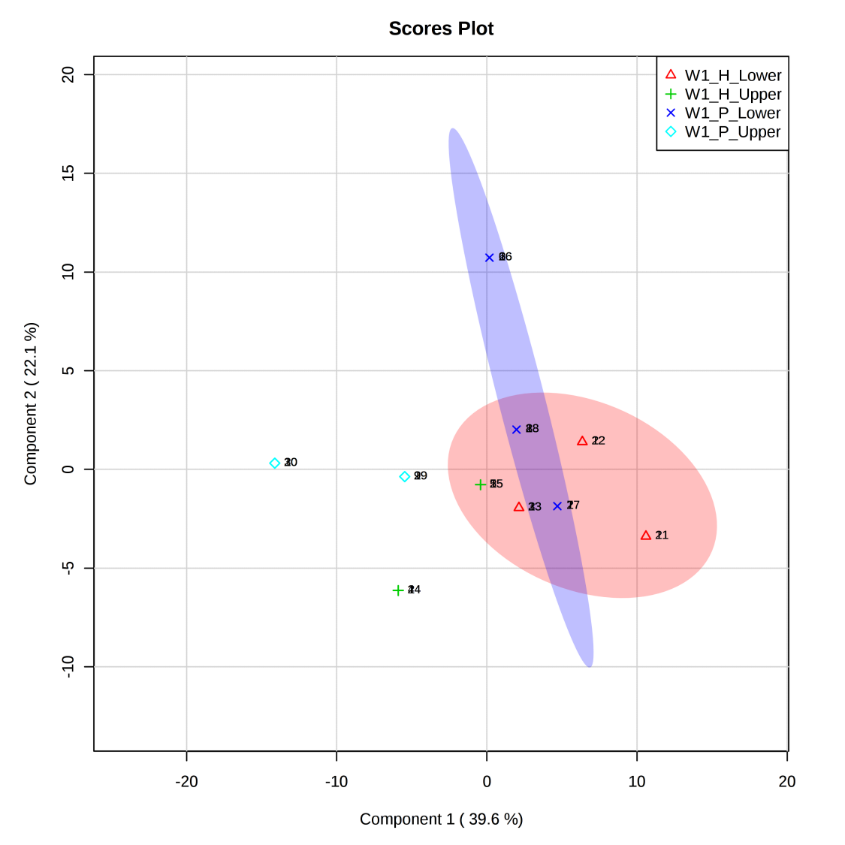


**Figure S4.** PLSDA plot showing differences in metabolite profiles between the lower undisturbed soils (red triangles and dark blue Xs) and upper disturbed subsoil plots (green crosses and blue diamonds) on week 1 of the winter trial.


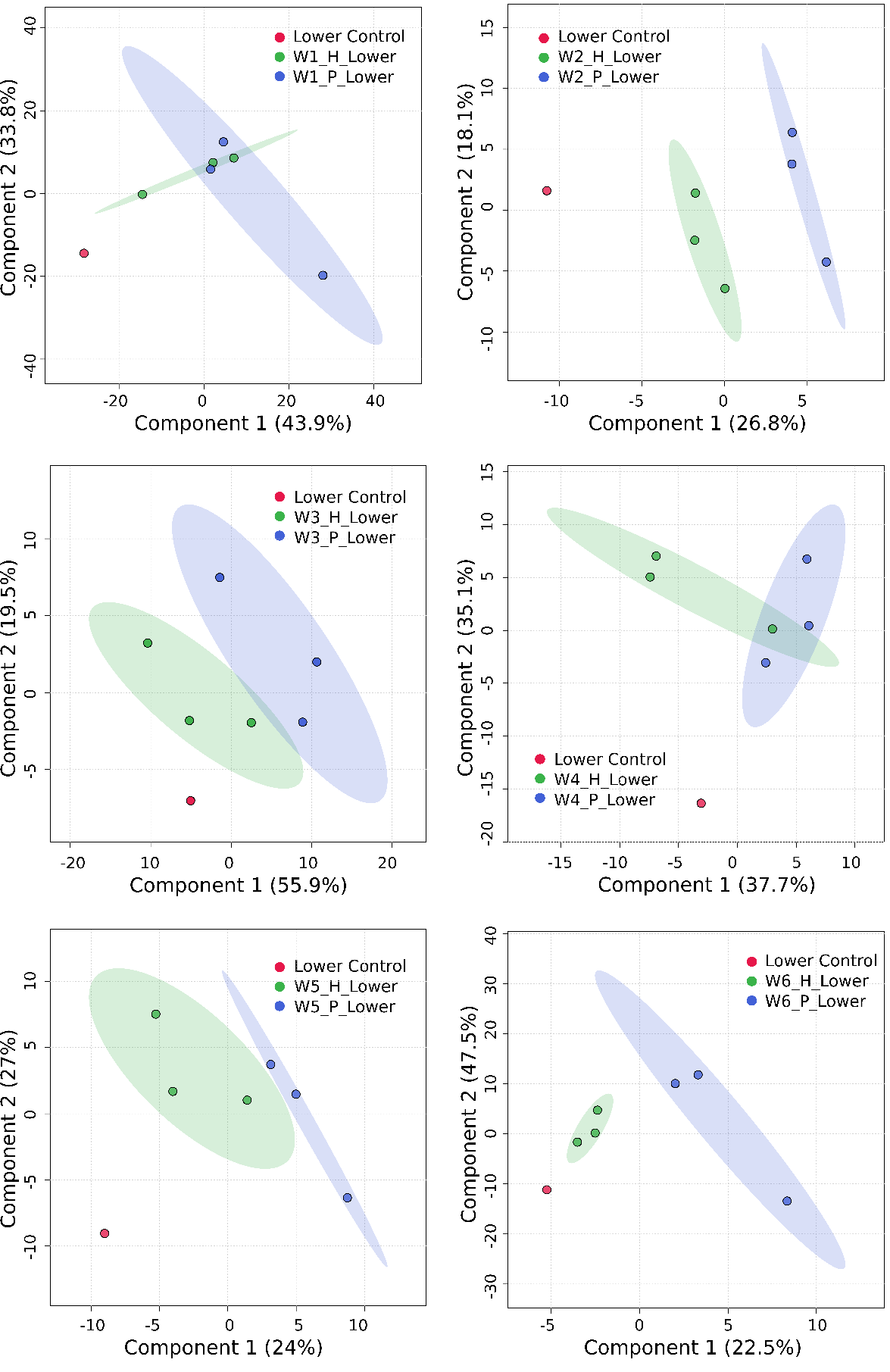


**Figure S5.** PLSDA plots showing differences in metabolite profiles between human decomposition (green), pig decomposition (purple) and no decomposition control (red) soils over the first six weeks (W1 through W6) of the winter trail at the lower location. Hotelling's ellipses in PLS-DA plots denote 95% confidence intervals. Upper site showed similar separation between humans, pigs and controls starting at Week 2 (data not shown).

**
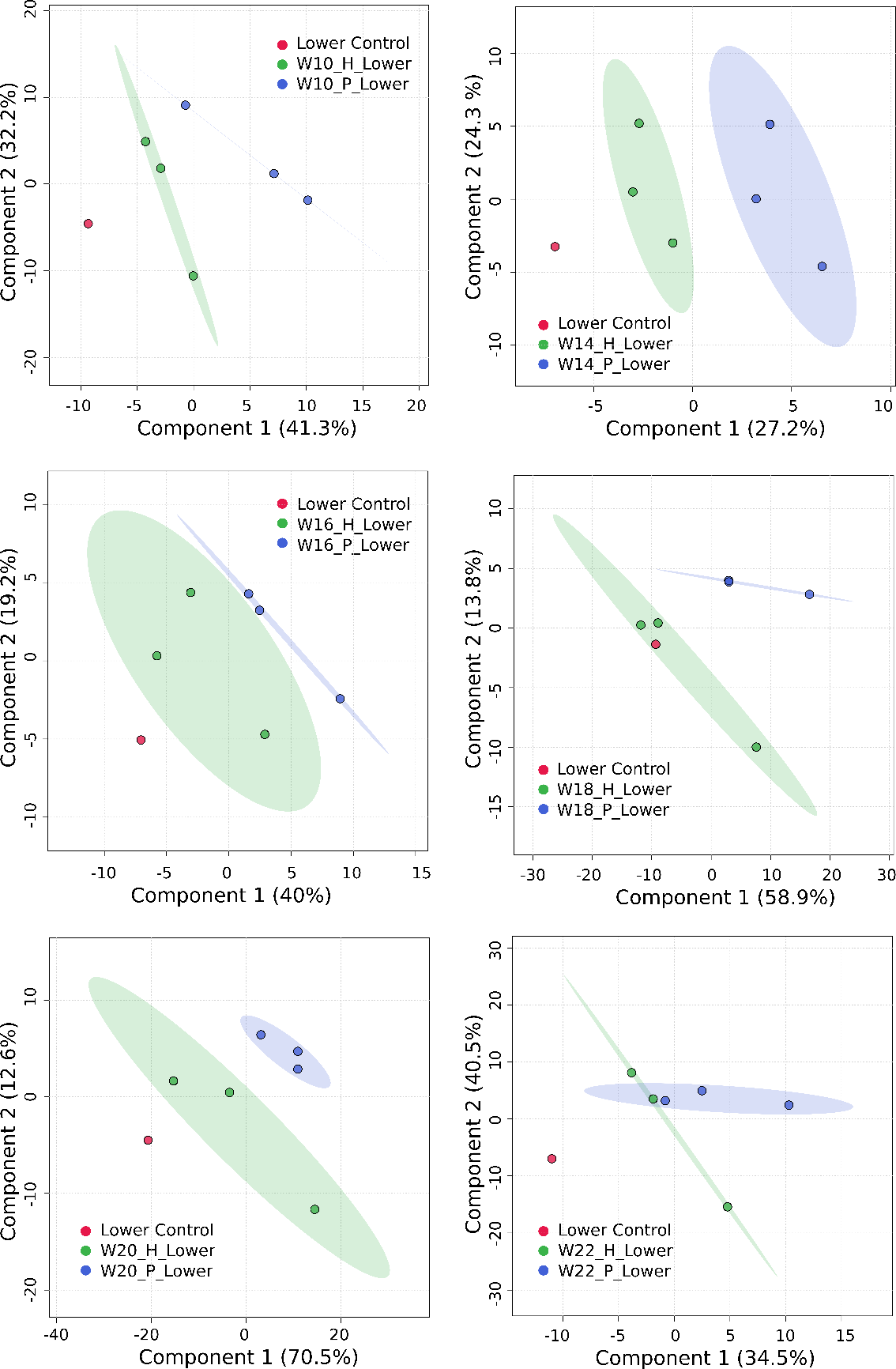
**

**Figure S6.** PLSDA plot showing differences in metabolite profiles between human decomposition (green), pig decomposition (purple) and no decomposition control (red) soils from week 10 to 22 (W10 through W22) of the winter trial at the lower location. Hotelling's ellipses in PLS-DA plots denote 95% confidence intervals. Upper site showed similar separation between humans, pigs and controls starting at Week 2 (data not shown).


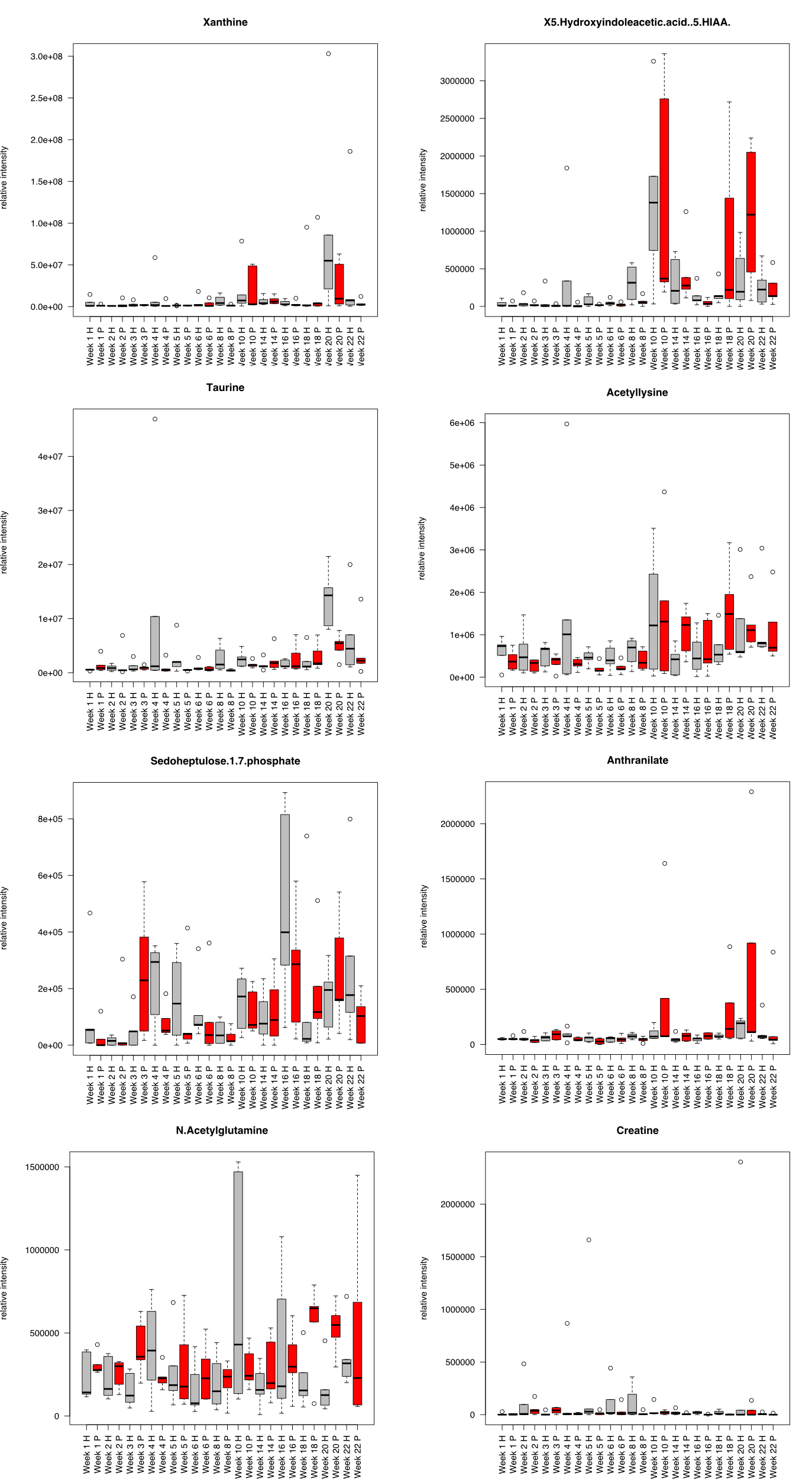

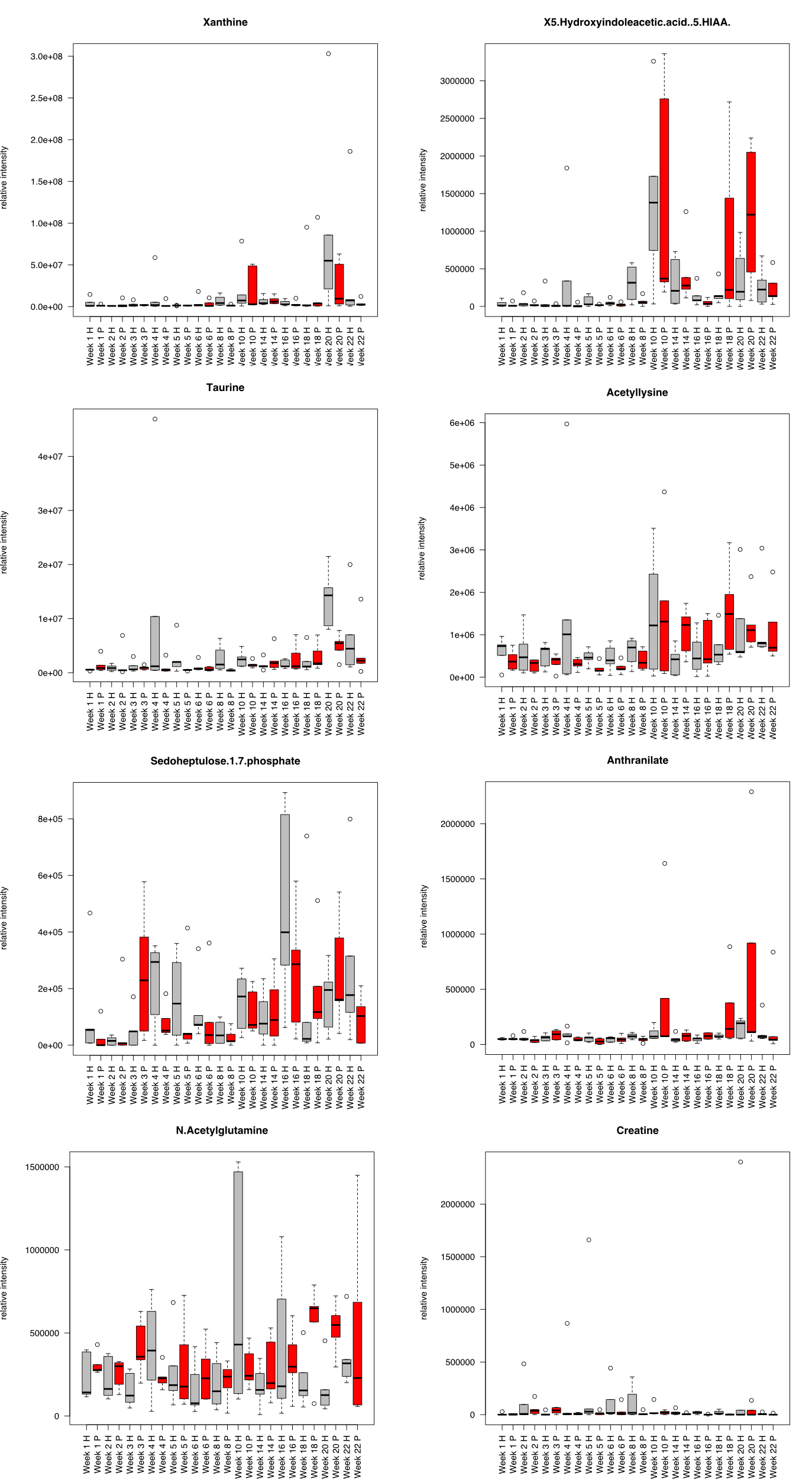

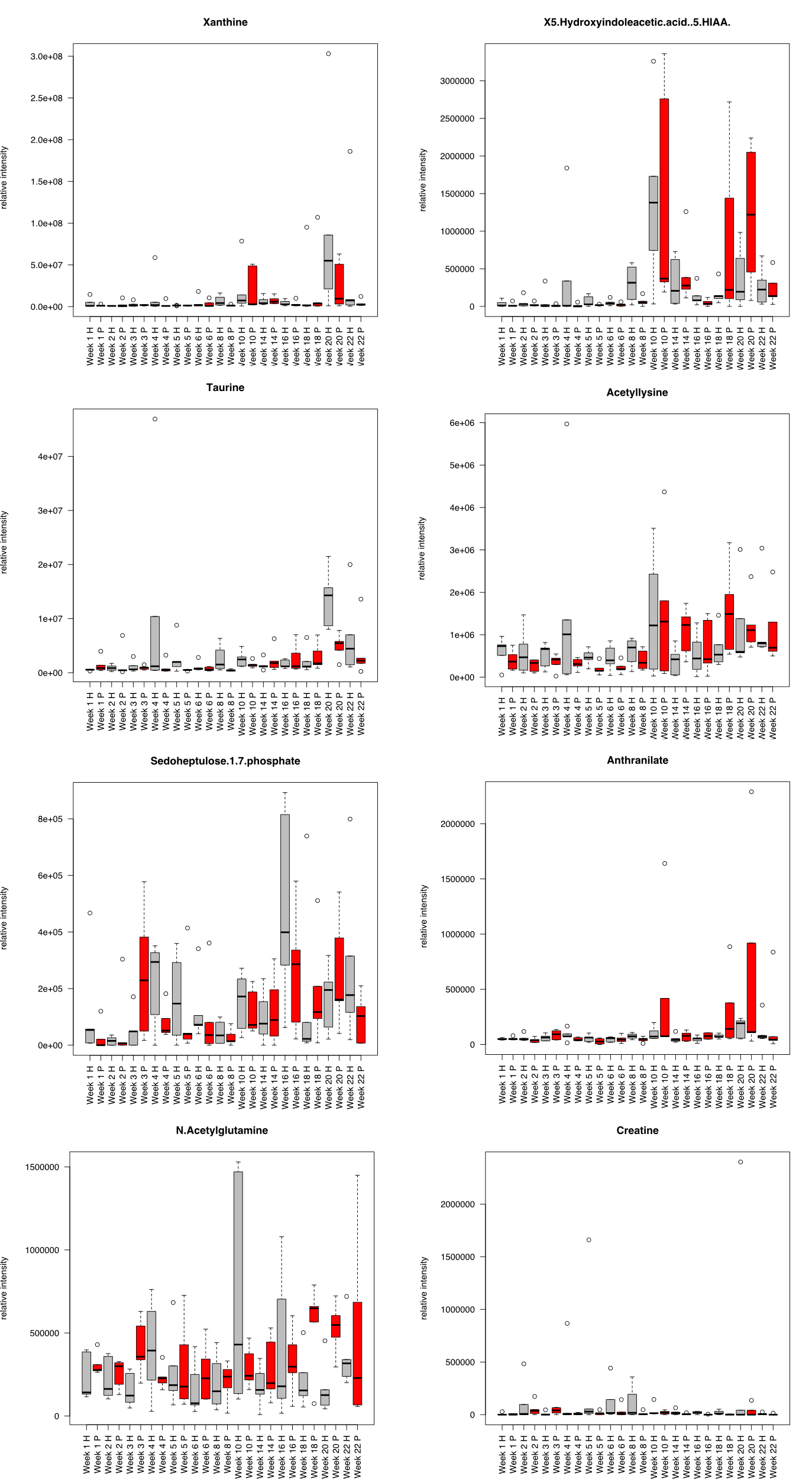


**Figure S7.** Mean relative intensities of metabolites most frequently detected in decomposition soils of humans (H, grey) and pigs (P, red) during the winter trial.
